# Long-Term BCI Training of a Tetraplegic User: Adaptive Riemannian Classifiers and User Training

**DOI:** 10.3389/fnhum.2021.635653

**Published:** 2021-03-18

**Authors:** Camille Benaroch, Khadijeh Sadatnejad, Aline Roc, Aurélien Appriou, Thibaut Monseigne, Smeety Pramij, Jelena Mladenovic, Léa Pillette, Camille Jeunet, Fabien Lotte

**Affiliations:** ^1^Inria Bordeaux Sud-Ouest, Talence, France; ^2^LaBRI (CNRS, Univ. Bordeaux, Bordeaux INP), Talence, France; ^3^CLLE Lab, CNRS, Univ. Toulouse Jean Jaurès, Toulouse, France

**Keywords:** brain computer interface, user training, electroencaphlography, Riemannian classification, tetraplegic or quadriplegic people, adaptive classification, user experience, learning metrics

## Abstract

While often presented as promising assistive technologies for motor-impaired users, electroencephalography (EEG)-based Brain-Computer Interfaces (BCIs) remain barely used outside laboratories due to low reliability in real-life conditions. There is thus a need to design long-term reliable BCIs that can be used outside-of-the-lab by end-users, e.g., severely motor-impaired ones. Therefore, we propose and evaluate the design of a multi-class Mental Task (MT)-based BCI for longitudinal training (20 sessions over 3 months) of a tetraplegic user for the CYBATHLON BCI series 2019. In this BCI championship, tetraplegic pilots are mentally driving a virtual car in a racing video game. We aimed at combining a progressive user MT-BCI training with a newly designed machine learning pipeline based on adaptive Riemannian classifiers shown to be promising for real-life applications. We followed a two step training process: the first 11 sessions served to train the user to control a 2-class MT-BCI by performing either two cognitive tasks (REST and MENTAL SUBTRACTION) or two motor-imagery tasks (LEFT-HAND and RIGHT-HAND). The second training step (9 remaining sessions) applied an adaptive, session-independent Riemannian classifier that combined all 4 MT classes used before. Moreover, as our Riemannian classifier was incrementally updated in an unsupervised way it would capture both within and between-session non-stationarity. Experimental evidences confirm the effectiveness of this approach. Namely, the classification accuracy improved by about 30% at the end of the training compared to initial sessions. We also studied the neural correlates of this performance improvement. Using a newly proposed BCI user learning metric, we could show our user learned to improve his BCI control by producing EEG signals matching increasingly more the BCI classifier training data distribution, rather than by improving his EEG class discrimination. However, the resulting improvement was effective only on synchronous (cue-based) BCI and it did not translate into improved CYBATHLON BCI game performances. For the sake of overcoming this in the future, we unveil possible reasons for these limited gaming performances and identify a number of promising future research directions. Importantly, we also report on the evolution of the user's neurophysiological patterns and user experience throughout the BCI training and competition.

## 1. Introduction

A Brain-Computer Interface (BCI) processes a user's brain activity often measured using Electroencephalography (EEG), and translates it into commands for an interactive application (Clerc et al., 2016). In this work, we particularly focus on Mental-Task based BCIs (MT-BCIs) that make use of mental-tasks (e.g., imagined movements or mental calculations) to control the system. Although promising, BCIs remain barely used outside laboratories notably due to their low reliability (Clerc et al., 2016): the average performance of MT-BCI users is most of the time rather low, e.g., around 75% of classification accuracy for 2-class motor imagery-BCIs on naive users (Guger et al., 2003; Blankertz et al., 2010). Furthermore, only a small number of studies focused on BCI end-users (e.g., users with severe motor impairment, Kauhanen et al., 2007; Conradi et al., 2009; Daly et al., 2013) in real-life testing environments outside the labs (Brandl et al., 2015; Statthaler et al., 2017; Perdikis et al., 2018; Perdikis and Millan, 2020). It is in this context that we decided to participate in the CYBATHLON BCI series competition in Graz in 2019. The CYBATHLON is a multi-discipline international competition aiming at benchmarking and evaluating different assistive technologies on end users as well as showcasing them to the general public (Novak et al., 2018). The CYBATHLON was organized first by the Swiss federal institute of technology in Zurich in 2016 and then again in 2020. This event consists of races in 6 disciplines (functional electrical stimulation bike, powered arm prosthesis, powered leg prosthesis, powered exoskeleton, powered wheelchair, and BCI) with different challenges. The CYBATHLON series is a spin off of the main event that focuses on each of those disciplines. The CYBATHLON BCI series 2019, that we participated in, was held in Graz (Austria) alongside the 8th International Graz Brain-Computer Interface conference, therefore allowing the racing teams to present their technologies and methods to the whole BCI community. For this event, a computer racing game mimicking a real-life application was designed (Novak et al., 2018). In this BCI game, tetraplegic pilots are asked to use up to four mental commands of their choice to control a virtual car.

The training of our pilot for that competition was divided into three parts in which we made adjustments in terms of learning for both the pilot and the machine. We started with an *exploratory phase*, including runs of closed-loop 2-class MT-BCI practices, where both the trainers and the trainee could apprehend the challenges, and agree upon a training routine. This was followed by a progressive training, using first a *2-class MT-BCI training phase* and then *a transfer phase* including 4-class MT-BCI training in both the standard minimalist training environment (used in previous phases) and the actual racing game environment. A progressive training seem essential for BCI as its efficiency relies on the users' ability to produce EEG patterns that are stable over time and distinct between the different mental commands (McFarland et al., 2010; Chavarriaga et al., 2016). Although improving users' ability to produce such signals through user training can certainly help the participants in controlling MT-BCI (Lotte and Jeunet, 2015; Perdikis and Millan, 2020; Roc et al., 2020), various sources of variability can lead to large shifts of data distribution between different sessions and consequently between the BCI classifier testing and training sets. Beside the largely unknown phenomena in the activity of neuronal populations which lead to non-stationarity of EEG signal (Kaplan et al., 2005), some variability sources including various environmental noises and changes in users' mental states such as their attention, fatigue or stress level are expected in an actual practice such as the CYBATHLON competition. When using a classifier trained on data from previous days (to avoid spending time on calibration on a new day), it tends to produce data shift between training and test sets/sessions, and thus create a BCI that is neither robust nor reliable (Shenoy et al., 2006; Li et al., 2010).

A recent signal processing approach which won multiple BCI challenges is based on the Riemannian geometry of covariance matrices (Congedo et al., 2017; Yger et al., 2017). This approach consists in describing EEG trials through spatial covariance matrices and analyzing them in a Riemannian framework (see section 2.4.2.2). Its unique properties led to a successful analysis of noisy data which contained many outliers (Yamamoto et al., 2020). Such framework does not require much training data nor the typical BCI spatial filtering data optimization to achieve high performances (Congedo et al., 2017). This motivated us to choose the Riemannian classifier for our machine learning pipeline.

Major issues in BCI signal processing include non-stationarities or signal variabilities which can be caused from e.g., changeable user skills or states (Mladenović et al., 2017). These variabilities could be addressed by adaptive learning techniques in BCI (Shenoy et al., 2006; Mladenović et al., 2017; Lotte et al., 2018). The strength of adaptive approaches in accommodating non-stationarity led to their superiority in both online and offline BCI experiments (Lotte et al., 2018). BCI performance could also be significantly improved by combining analysis in a Riemannian framework of covariance matrices with adaptive techniques to address the omnipresence of non-stationarity (Kumar et al., 2019). Note that such work only addressed within-session variabilities, but not necessarily between session ones. To address between-session non-stationarity effects, we propose a new method that projects all sessions to a common reference, by matching the geometric mean of a few minutes of EEG signals collected at the beginning of each session.

In this work, we address both long-term BCI user training with a BCI-naive tetraplegic user, and out-of-the-lab BCI use across multiple sessions, thus facing various non-stationarity problems. We present our approach that combines a progressive MT-BCI user training and a new adaptive Riemannian classification method that can model both within and between-session variability. Furthermore, we report on the evolution of the CYBATHLON pilot BCI classification performances, neurophysiological patterns and User eXperience (UX) along the training sessions. Regarding the study of neurophysiological patterns of user learning, beside the typical metrics, we propose a new metric to measure user learning in terms of how much the user adapts his/her EEG signals to the BCI classifier training set. Interestingly enough such metric could reveal a new form of BCI user learning. Finally, we also reflect on the pros and cons of this approach, in order to identify future areas of improvement.

This paper is organized as follows: section 2 presents the methods designed and employed for: user training, UX evaluation, EEG signal processing and machine learning and neurophysiological signal analyses. Then, section 3 presents the results obtained along the training and at the competition in terms of classification performances, EEG patterns and UX. Section 4 discusses those results, while section 5 draws lessons from them for future works. Finally, section 6 concludes the paper.

## 2. Methods

As the CYBATHLON BCI series 2019 was a first experience for both the pilot and the research team, during the first 7 sessions we explored a suitable set of mental tasks and EEG sensors to use. We called this period the *exploratory phase*. Then, we progressively trained both the pilot and the machine to increase the ability of the user to generate stable and distinct brain signals for each selected task. Finally, the pilot was able to train with the racing game in which the completion time was used to evaluate performance. In the following section, we will describe our pilot as well as the protocol in each phase of the training.

### 2.1. Pilot

Our pilot, a French male who was 32 year-old at the time of the study, was injured in 2007. He has a level-C4 spinal cord injury, with an “A” score (Complete injury — No motor or sensory function is preserved in the sacral segments S4 or S5) on the ASIA impairment scale. He has a residual left shoulder motor ability. The pilot was a naive BCI user when we started the CYBATHLON training. The study was approved by the Inria ethical committee, the COERLE (approval number 2019-12), and the pilot signed an informed consent form. The training took place between June and September 2019.

### 2.2. User Training

#### 2.2.1. The Objective: The CYBATHLON BCI Race

For the CYBATHLON race, each driver sits in front of a separate screen to play the game by controlling a racing vehicle avatar. Pilots can visualize the other players on separate tracks below their own. The driver whose avatar crosses the finish line first wins the race (i.e., the race completion time is the criterion for evaluating the pilot's performance).

**Figure 1:**
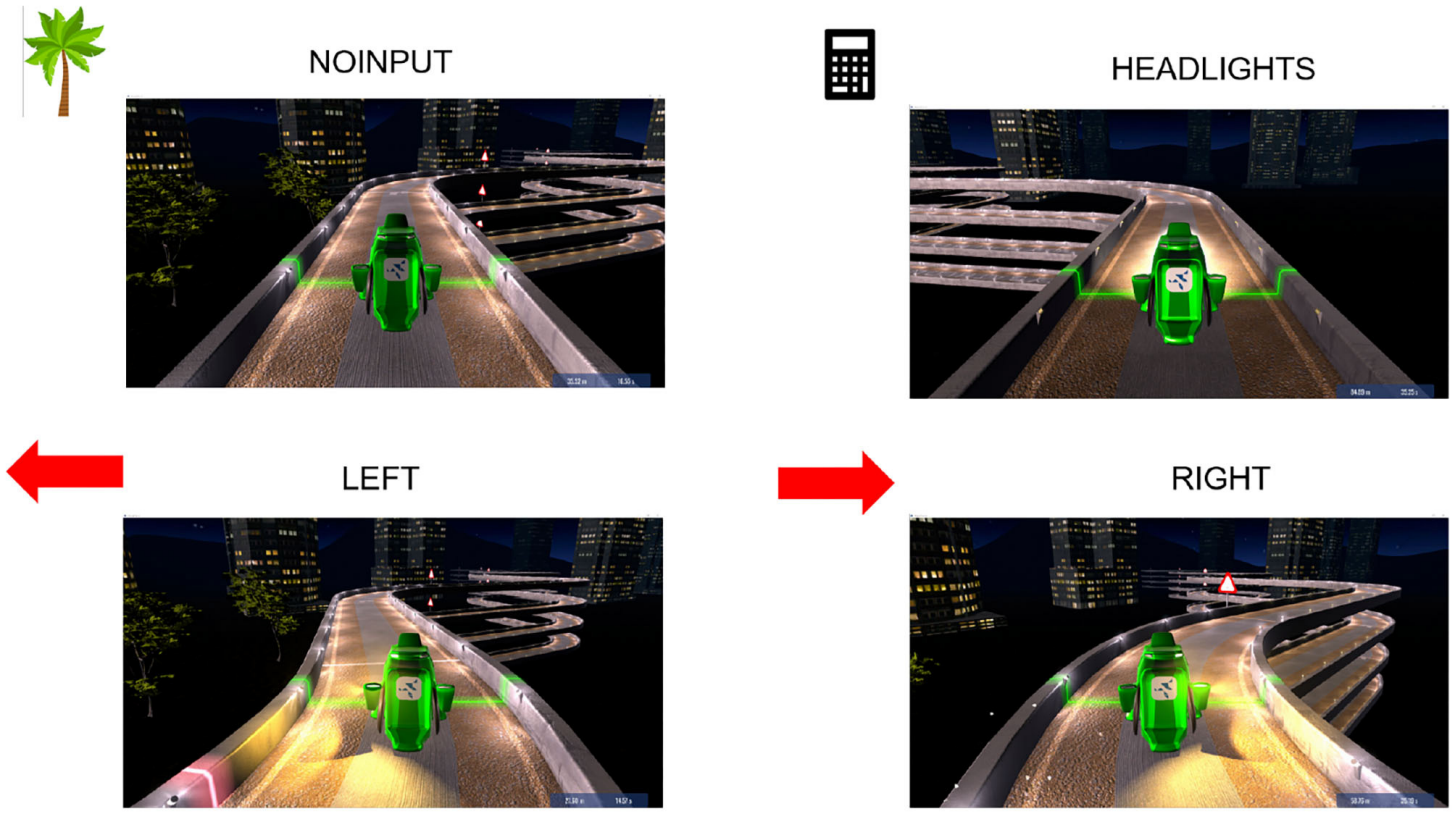
CYBATHLON racing game. We chose a palm tree as the pictogram for the REST state (i.e., the NOINPUT command), a calculator for the MENTAL SUBTRACTION task (i.e., HEADLIGHTS command), and arrows pointing to the left and right for, respectively, LEFT and RIGHT-HAND motor imagery (i.e., turning LEFT and RIGHT commands).

To control the vehicle, the pilot can modulate his EEG signals to send commands with the BCI. Depending on the course of the track, pilots can trigger three active commands (“LEFT,” “RIGHT,” “HEADLIGHTS”), to, respectively, make the car turn left, turn right or switch the headlights on, or they can just trigger the default command (“NOINPUT”), which makes the vehicle move by itself even when no input signal is sent. Figure 1 shows the four instructions of the game (i.e., “LEFT,” “RIGHT,” “HEADLIGHTS,” and “NOINPUT”) and their corresponding pictograms used during the user training. Indeed, before training with the game, the user trained with a classic BCI paradigm (see **Figure 4** for the paradigm).

The vehicle accelerates during the time windows where the right command is sent. A wrong command sent by accident results in a disadvantage, i.e., the avatar slows down and loses time (e.g., “HEADLIGHTS” instead of “RIGHT,” or “LEFT” instead of “NOINPUT”). The method we used to decide whether or not to send a command based on the classification outputs is detailed in section 2.4.2.3.

#### 2.2.2. Training Schedule

Sessions typically started in the morning, taking place at the pilot's home and on a few occasions in the laboratory (for sessions 10, 12, 13, 16, and 19). Two to three researchers among this paper authors were always present. The pilot training lasted approximately 3 months, between June 2019 and September 2019, and comprised a total of 20 training sessions. The training schedule was flexible, ranging from one to three training sessions a week, and from one to 2 h a session. All these 20 sessions included closed-loop training runs with online BCI feedback.

**Figure 2:**
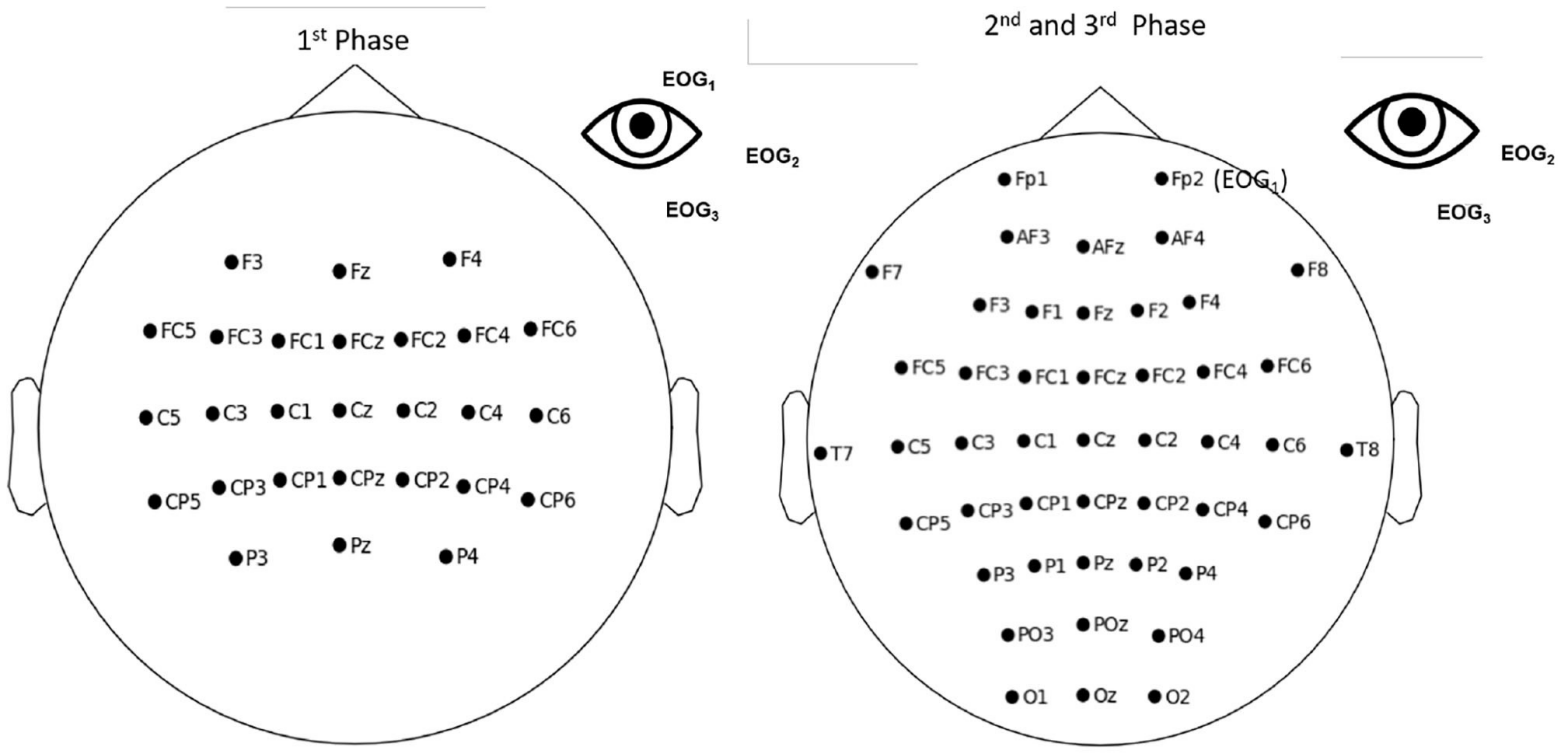
Electrodes used during the first (exploratory) phase **(left)**, and the second (2-class training) and third (transfer) phases of the training **(right)**. For all the phases we used the same EEG hardware g.USBAmp (g.tec, Austria), sampled at 512 Hz) but we added more electrodes (16) for the second and third phase.

The training was divided into three phases:

- **The exploratory phase**:
  A phase of familiarization and screening where both the trainers and the trainee could apprehend the challenge and set up a training routine (7 sessions). During this phase, several decisions had to be taken such as the Mental Tasks (MT) the pilot had to perform so that the designed BCI can send one of the four possible commands. From the first sessions, our pilot showed an interest in performing motor imagery. Therefore, motor imagery of the RIGHT-HAND and LEFT-HAND were included. Those were later associated with the controls to make the game vehicle turn right and left, respectively, to ensure a clear mapping between the MT and their effect. The rest state was included as the “NOINPUT” command and the last task (“HEADLIGHTS”) had yet to be settled. As the literature suggests that a multiclass BCI benefits from selecting user-specific tasks and mixing motor and cognitive MTs (Friedrich et al., 2013), both MENTAL SUBTRACTION and MENTAL ROTATION were screened for the last task. MENTAL SUBTRACTION was chosen as our pilot felt more comfortable with this task. This phase enabled the pilot to apprehend the practice of Mental Tasks and to familiarize himself with the online feedback provided by the BCI classifier. During each of these sessions, he practiced in closed-loop with a 2-class MT-BCI whose classifier was trained on the data of the two first runs of that session. This phase also enabled us, the experimenters, to identify which MTs to train in a more systematic and controlled way in the subsequent phase.
- **The 2-class progressive training phase**:
  An intermediate phase that is part of a progressive training. Indeed, the literature on human learning suggests that it is best to train components of the task before the complete task (Merrill, 2007). Progressive training has previously attained high BCI performances (McFarland et al., 2010). Thus, we trained the pilot with a subset of the mental tasks before moving up to the full 4-class control. The pilot was trained to control a 2-commands BCI during 4 sessions (see **Figure 2** for the paradigm). The training involved two different pairs of commands: two sessions were dedicated to LEFT- vs. RIGHT-HAND movement imagination (7 runs in total) and two sessions to MENTAL SUBTRACTION vs. REST (6 runs in total). The tasks and the number of electrodes were fixed.
- **The transfer phase**:
  A final training phase where the pilot alternated between 4-class online BCI and the actual CYBATHLON racing game training (hence the phase name). The game version delivered to the contestants was used for the training and then modified with random instructions order for the command, so that each race is different (only the order of the instructions during the race was changed). This phase lasted 9 sessions. The pilot started to train with the actual CYBATHLON racing game version at session 13. He was able to experience the race during 7 sessions (30 races).

#### 2.2.3. BCI Protocol

At the beginning of each session, the pilot had to complete a short (about 5 min) questionnaire about his current state, and then the EEG cap was installed (about 20 min) while experimenters were informally chatting with the pilot. After the cap installation, two EEG baselines (resting state) were recorded, respectively, with eyes open and eyes closed (2*3 min).

To record the EEG signals, a different number of active scalp electrodes, referenced to the left ear, were used in the three training phases (see Figure 2). EEG signals were measured using a g.USBAmp (g.tec, Austria), sampled at 512 Hz and processed online using OpenViBE 2.2.0. (Renard et al., 2010). We decided to increase the number of electrodes between the exploratory and 2-class training phase. Contrary to the setup used in the exploratory phase that only used motor-related electrodes, the additional electrodes served mainly for MENTAL SUBTRACTION and REST MT.

For the exploratory phase, we used the standard “Graz BCI” bar feedback (Pfurtscheller and Neuper, 2001), as implemented in OpenViBE for 2-class MT-BCIs and as used in, e.g., Roc et al., 2019; Pillette et al., 2021. In that phase each run included 20 trials for each of the two MT classes. A classifier was built on the data from the two first runs to provide online feedback for the subsequent training runs of that session. Due to the exploratory and non-systematic nature of that phase, the number of runs performed fluctuated largely between sessions.

**Figure 3:**
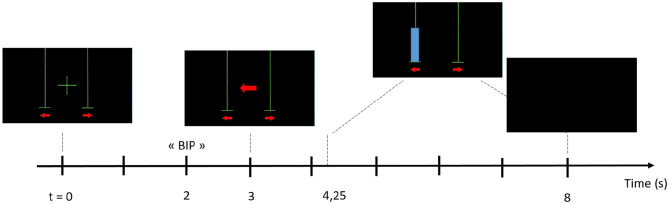
Experimental paradigm used for the 2 classes MT-BCI and more specifically here for the 2-class motor imagery training (LEFT-HAND vs. RIGHT-HAND motor imagery).

**Figure 4:**
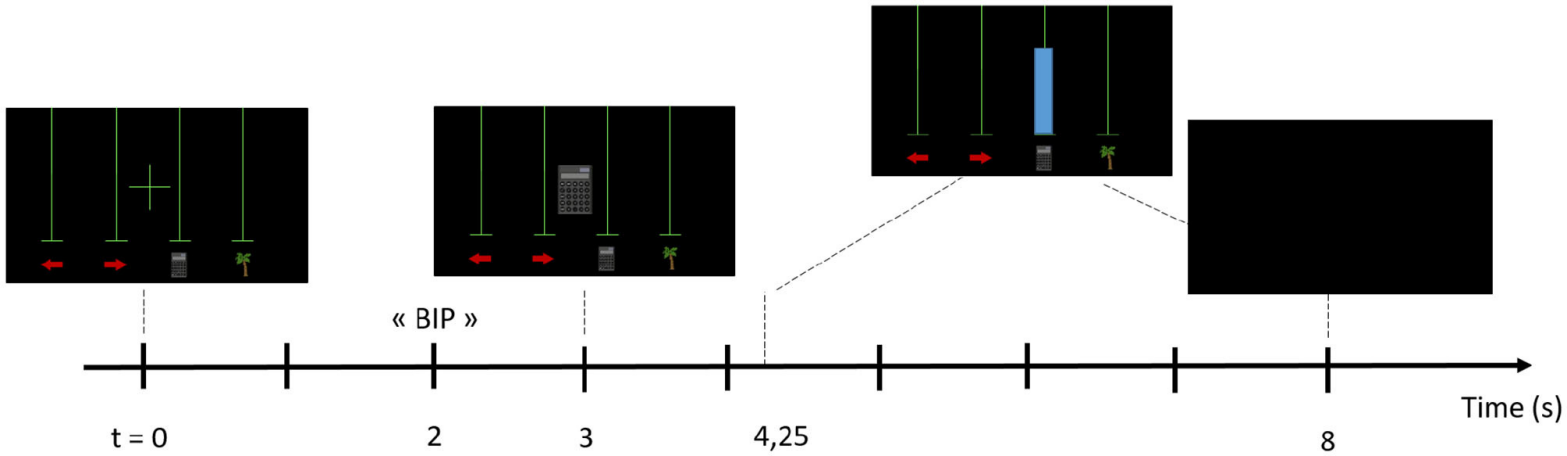
Experimental paradigm used for the 4 classes MT-BCI.

The same paradigm was used for all online BCI runs during the 2-class training phase (see Figure 3). Each run comprised 40 trials (i.e., 20 trials per class). The number of runs depended on the session duration and the pilot's state, notably his motivation and fatigue. For each trial, a cross was first displayed. The Mental Task (MT) to be performed was then announced by a “beep” and the corresponding pictogram appeared in the middle of the screen, i.e., an arrow pointing to the left representing a LEFT-HAND motor imagery task; an arrow pointing to the right representing a RIGHT-HAND motor imagery task; a calculator representing a MENTAL SUBTRACTION task; a palm tree representing the REST state, chosen together with the pilot. Then, a blue bar was displayed as continuous visual feedback. The location of this bar (i.e., over which pictogram it appears) indicated the MT recognized by the classifier and its length the classifier confidence in this recognition. The bar was displayed only when there was a match between the instruction and the recognized task, i.e., it was a positive only feedback. During the training phase, we alternated between 2-class and 4-class MT. Hence, we used the same paradigm for the 4-class phase (Figure 4). Each run comprised 40 trials (i.e., 10 trials per class) but this time the pilot had to perform one of the four chosen tasks for the game.

After the cap removal, the majority of the sessions ended with a quick end-of-session questionnaire on the computer (about 5 min) and an informal debriefing concerning the mental strategy used to perform the tasks and, in response to the pilot's inquiries, the performances achieved, the meaning of the feedback or the classification method used.

### 2.3. User Experience

#### 2.3.1. Mental Tasks

The four tasks that were used are those described in Figure 1, they were chosen according to the pilot's wishes and a short screening. Regarding the Motor Imagery (MI) tasks associated with paralyzed limbs [i.e., Left-Hand (LH) and Righ-Hand (RH)], the pilot experimented with several options to determine what he felt most comfortable with (e.g., imagination of opening and closing his hand) and finally settled on the imagination of boxing moves for each hand. The third Mental Task (MT), i.e., MENTAL-SUBTRACTION (MS) task, was initially conducted with cues in the exploratory phase, where the pilot was shown 3-digit numbers on the screen and was instructed to gradually subtract randomly generated two-digit numbers from them. Then, in the 2-class BCI training phase and the transfer phase, the pilot could perform mental math without cue, i.e., he was instructed to spontaneously choose a “random” number and to make the corresponding subtractions of 2-digit numbers by 1-digit numbers. He felt comfortable with this third task.

#### 2.3.2. Questionnaires

Before and after most of the sessions, our pilot completed questionnaires regarding his subjective states (see Appendix A in Bismuth et al., 2020). Both questionnaires retrieved user experience (UX-) related factors based on subjective 5-point Likert scale in 8 items (pre-session) and 21 items (post-session). Based on these items, a score can be computed for 5 factors, i.e., mood, motivation, and mindfulness (assessed pre- and post-session) along with post-session agency (feeling of control over the feedback provided by the system) and cognitive load (amount of cognitive process required to control the system).

#### 2.3.3. Interview

After the competition, an interview was conducted with the pilot. The aim was to analyse qualitatively the impact that the whole process (from the first contact, to the BCI training and competition) had on the pilot's representations related to BCIs. More precisely, using a semi-structured interview approach, we investigated the pilot's initial acceptability of BCIs, including different dimensions such as motivation, perceived usefulness, perceived ease of use and intention to use BCI-based technologies. Then, we sought to understand how his CYBATHLON experience modified these representations, and what level of acceptance resulted from this “adventure.” Due to the COVID-19 crisis, the interview was conducted remotely (video conference). It lasted 1 h and started with an explanation of the objectives and structure of the conversation to come. It was divided into four parts dedicated to different moments: (i) before the BCI training, (ii) during the BCI training, (iii) during the CYBATHLON competition, and (iv) after the CYBATHLON competition. Each part was divided into a series of questions related to different dimensions of acceptability and acceptance, mainly based on the Technology-Acceptance Model (TAM) 3 (Venkatesh and Bala, 2008) questionnaire. The TAM3 suggests that one's usage behavior is determined by their usage intention, itself being influenced by both the perceived usefulness and perceived ease of use of the technology. According to the TAM3, the perceived usefulness and ease of use would be influenced by social norms, inter-individual differences (i.e., psychological traits and states and socio-demographic characteristics), facilitating conditions and characteristics related to the technology. The influence of these factors would be modulated by the fact that the technology use is voluntary or not, and by the experience that the user has with the technology.

### 2.4. Signal Processing and Machine Learning

Due to the exploratory, and non-formal nature of this work (a competition preparation), the machine learning tools we used evolved along with the training, according to the problems we encountered. During the exploratory phase, we first started with a standard MT classification pipeline based on Common Spatial Pattern (CSP) spatial filtering (Ramoser et al., 2000) and a Linear Discriminant Analysis (LDA) classifier (Duda et al., 2012), for the exploratory phase. For this phase, we calibrated the CSP and LDA on the first two runs (acquisition runs) of each session. Due to sensitivity of CSP to noise and outliers (Reuderink and Poel, 2008; Arvaneh et al., 2012), and since we performed user training mostly at home, an environment with different variability sources, a more robust approach was used for the next phase. During the 2-class training phase, we thus represented EEG by spatial covariance matrices and analyzed them in a Riemannian framework (Congedo et al., 2017; Yger et al., 2017). Indeed, such Riemannian classifier proved very efficient for EEG signal classification in several offline EEG classification competitions (Congedo et al., 2017; Yger et al., 2017). In this phase, we also calibrated the Riemannian classifier on each session acquisition runs.

For the competition day, we needed a previously trained classifier to avoid calibration time. Moreover, recalibrating the classifier everyday could lead to an ever-changing feedback which may be detrimental to user training (Perdikis et al., 2018; Perdikis and Millan, 2020; Roc et al., 2020). Therefore, for the transfer phase (session 12 and subsequent) we calibrated our classifier based on the runs from the previous phase sessions that were the least noisy and the least contaminated by artifacts. Nonetheless, for the two first sessions of the transfer phase, considerable shifts and performance variation between sessions were observed. Therefore, we started using an adaptive approach from session 14 onward. This approach consisted in reducing non-stationarity notably by projecting all training sessions data into a common subspace, and then in adaptively projecting the test set to this common subspace. We describe the technical details of the preprocessing, features, classifiers, adaptation method, inter-session variability reduction, and evaluation criteria in this section.

#### 2.4.1. Preprocessing

During the first two phases where the classifier was trained on the data of dedicated in-session runs, EEG signals were processed by segmenting them into 1 s windows starting from 1.25 s after the start of the instruction cue, with 93.75% overlapping (i.e., 1/16 s shift) both for training the classifier and online-test. During the last phase (i.e., transfer), since we started using multiple training sessions, different overlaps were used for the classifier calibration and for the online classification. For the calibration of the classifier, notably for computational and memory reasons, we chose smaller overlaps between consecutive windows (between 50 and 87.5% depending on the classifier). For online evaluation, since a continuous feedback was needed, we used 93.75% overlapping between consecutive windows for session 12 and 13 and 87.5% from session 14 and for the game. All EEG signals were band-pass filtered in 8–24 Hz using a butterworth filter of order 4.

Under the CYBATHLON BCI race regulation, some preprocessing was applied to ensure that the pilot controls the avatar using signals originating solely from brain activity and not signals of a muscular origin. The main goal of this preprocessing was rejecting artifacts. We rejected all EEG epochs in which band-pass filtered EEG had absolute amplitude higher than 70 μV, or included horizontal or vertical eye movements. To detect eye-movement related artifact, three electrodes were put around the left eye, i.e., EOG1, EOG2, and EOG3. Vertical eye movements were detected with EOG1 and EOG3 put on the vertical line of the eye, up and down, respectively. EOG2 was put on the horizontal line of the eye. The electrodes placement is shown in Figure 2. The EOG signals were filtered in 1–10 Hz using 4-order butterworth filter. To detect the possible eye artifacts, we computed two vertical and horizontal signals as follows:

(1) $$\begin{array}{r} V=EOG1-EOG3 \end{array}$$

(2) $$\begin{array}{r} H=EOG2-\frac{\left(EOG1+EOG3\right)}{2} \end{array}$$

where *V* and *H* are related to vertical and horizontal eye movements, respectively. After preprocessing, each EEG window that did not include a local peak of vertical or horizontal EOG was considered as clean and used for model training and online classification. Otherwise, it was rejected. In order to find the local peaks of these signals, thresholds were applied over the average absolute value of the vertical/horizontal signals in each window as follows:

(3) $$\begin{array}{r} \frac{1}{T}\times \overset{t=T}{\underset{t=1}{\sum }}|V_{t}|<T_{V} \end{array}$$

(4) $$\begin{array}{r} \frac{1}{T}\times \overset{t=T}{\underset{t=1}{\sum }}|H_{t}|<T_{H} \end{array}$$

where *T*_*V*_ and *T*_*H*_ denote the thresholds for finding vertical and horizontal eye movement, *T* denotes the time length of each window, *V*_*t*_ and *H*_*t*_ denotes the vertical and horizontal signal at *t*^*th*^ time point of each window. The threshold values for vertical and horizontal signals were estimated on training data as follows:

(5) $$\begin{array}{r} T_{S}=\frac{\sum_{t=1}^{t=N}|S|}{N}+3\sigma_{\left|S\right|} \end{array}$$

where *S* could be either *V* or *H* signals, σ_|*S*|_ is the standard deviation of the signal |*S*|, and *N* denotes the time-length of the signal.

#### 2.4.2. Features and Classifiers

Due to the exploratory nature of this work we switched between two main online classification pipelines for MT-BCI between the exploratory and the 2-class user training phases. The details of the features and classifiers used online during the different training phases are as follows:

##### 2.4.2.1. CSP+LDA

In the exploratory phase, we used standard linear classifiers and spatial filters, to combine several channels (here with a linear combination) into a single one (Ramoser et al., 2000; Lotte, 2014). We notably used the Common Spatial Pattern (CSP), a popular approach that extract discriminative spatial filters for classification (Ramoser et al., 2000; Lotte, 2014). In our experiments, for the exploratory phase, we used 6 CSP filters, corresponding to the 3 largest and 3 smallest eigenvalues (Ramoser et al., 2000; Lotte, 2014). We then used Linear discriminant Analysis (LDA), a linear discriminative classifier, for classifying CSP-based features (Duda et al., 2012).

##### 2.4.2.2. Riemannian Classifiers

Due to the presence of different noise and artifact sources during the exploratory phase, and due to the sensitivity of CSP to them, we moved on to analysis in a Riemannian framework for the 2-class training phase and continued with it for the transfer phase. EEG analysis in a Riemannian framework consists in describing EEG epochs by their Spatial Covariance Matrices (*SCM*) and using Riemannian Geometry to consider the non-linear geometry of the space in analyses. In this section, we first define the principles of analysis in a Riemannian framework, then we describe the Riemannian classifiers we used. For describing the EEG epochs as SCMs, we used the optimal linear shrinkage estimator of SCM from Ledoit and Wolf (2004). We denote each SCM by $C_{j}^{\left(c_{i}\right)}$, where *c*_*i*_ is its corresponding class and *j* its epoch index. $C_{j}^{\left(c_{i}\right)}$ is an *N*×*N* Symmetric Positive Definite (SPD) matrix, where *N* denotes the number of EEG channels. The space of SPD matrices, which is not closed under scalar multiplication, is not a vector space. We reformulate the feature space as a Riemannian manifold by equipping each tangent space with a Riemannian metric. The Riemannian distance, which is the distance between two points (SCMs) along the manifold, can be computed as follows (Yger et al., 2017):

(6) $$\begin{array}{r} d_{R}\left(C_{i},C_{j}\right)=||log\left(C_{i}^{-1/2}\times C_{j}\times C_{i}^{-1/2}\right)|{|}_{F}={\left(\overset{N}{\underset{l=1}{\sum }}log^{2}\left(\lambda_{l}\right)\right)}^{1/2} \end{array}$$

where *i* and *j* are the indexes of the epochs, ||.||_*F*_ denotes the Frobenius norm, and λ_*l*_ is a positive eigenvalue of the $C_{i}^{-1/2}C_{j}C_{i}^{-1/2}$ matrix. By changing the analysis framework to a Riemannian framework, we needed to adapt geometrical and statistical concepts to this geometry. In the following, we only describe some definitions that we need for this paper.

**Riemannian Mean:** The mean points of SPD matrices, which is called Karcher/Frechet mean, is defined as a point over the manifold (i.e., an SPD matrix) which has minimum squared Riemannian distance from all the points over the manifold.

(7) $$\begin{array}{r} {\bar{C}}^{\left(c\right)}=argmin_{C}\overset{N_{c}}{\underset{i=1}{\sum }}d_{R}^{2}\left(C,C_{i}^{\left(c\right)}\right) \end{array}$$

where ${\bar{C}}^{\left(c\right)}$ denotes the geometric mean of *N*_*c*_ samples from class *c* (Lang, 2012). Since there is no closed form solution to this problem, we used the iterative estimator proposed in Moakher (2005).

**Riemannian variance**: The sample variance over the manifold, which is known as Frechet variance, represents the dispersion around the mean point over the manifold. We compute the sample variance as the expected value of the squared Riemannian distance around the geometric mean (Lang, 2012).

(8) $$\begin{array}{r} {\sigma^{\left(c\right)}}^{2}=\frac{1}{N_{c}}\times \overset{N_{c}}{\underset{i=1}{\sum }}d_{R}^{2}\left(C_{i}^{\left(c\right)},{\bar{C}}^{\left(c\right)}\right) \end{array}$$

**Fisher geodesic Minimum Distance to Mean** (FgMDM): FgMDM is a pipeline combining Fisher Geodesic Discriminant Analysis (FGDA) and Minimum Distance to Mean (MDM) classifier (Barachant et al., 2010). Geodesic filtering is achieved in tangent space with a Linear Discriminant Analysis to keep class-related information and discard irrelevant information. After filtering, data are projected back to the manifold and classified using a Minimum Distance to Means (MDM) classifier (Barachant et al., 2010). An MDM classifier works as follows: for training, it computes the Karcher mean of the training SCMs from each class. Then, for predicting the label of a test SCM, MDM computes the Riemannian distance of this SCM to each class mean and assigns it the label of the nearest class mean. The details of the FgMDM algorithm for training and testing can be seen in algorithms 1 and 2 of Appendix A, respectively.

**Adaptive rebias FgMDM:** As mentioned before, in order to reduce both within-session variability and shifts between training and test sets, we started using an adaptive approach in the transfer phase, from Session 14 onward. To do so, we used an adaptive rebias FgMDM classifier (Zanini et al., 2017; Kumar et al., 2019). Rebias FgMDM relies on aligning the covariance matrices from the training and test set onto a common reference. For this purpose, the idea is to identify a projector, one for the training set and one for the testing set, so that the Karcher mean of the projected SCMs for each set ends up on a common reference, here the identity matrix. With this approach, all SCMs $C_{i}^{\prime }$ are thus centered around the identity matrix, using the following transformation:

(9) $$\begin{array}{r} C_{i}^{\prime }={\bar{C}}^{-1/2}\times C_{i}\times {\bar{C}}^{-1/2} \end{array}$$

where $\bar{C}$ denotes the Karcher mean of the training/test set for projecting the training and test SCMs, respectively. Note that this projection does not require class labels, and is thus unsupervised. It should also be noted that when used online, the test set is not fully available, and thus the real $\bar{C}$ cannot be estimated. An adaptive estimation of it, based on incoming test SCMs, is thus used. More precisely, the test set Karcher mean is adaptively estimated using a weighted geodesic interpolation between previous estimates and each ongoing SCM, as follows:

(10) $$\begin{array}{r} {\bar{C}}_{i+1}={\bar{C}}_{i}^{1/2}\times {\left({\bar{C}}_{i}^{-1/2}\times C_{i+1}\times {\bar{C}}_{i}^{-1/2}\right)}^{1/\left(i+1\right)}\times {\bar{C}}_{i}^{1/2} \end{array}$$

where ${\bar{C}}_{i}$ is the current estimates of the test set Karcher mean, *C*_*i*+1_ denotes the ongoing SCM epoch, and ${\bar{C}}_{i+1}$ denotes the updated test set Karcher mean. ${\bar{C}}_{i+1}$ is then used to project the subsequent test SCMs using Equation (9). This estimate is initialized to the training set Karcher mean. See (Kumar et al., 2019) for more details on this adaptive rebias FgMDM. To the best of our knowledge, this is the first time that such an adaptive Riemannian classifier is used online for MT-BCI.

Whereas adaptive rebias FgMDM could reduce the shifts between the training and test sets as well as within-session variability, it does not address shifts and variability that may occur between sessions. However, our classifier is trained on multiple sessions, and aims at being applied unchanged on multiple sessions as well. We thus needed to address between-session variability as well.

**Figure 5:**
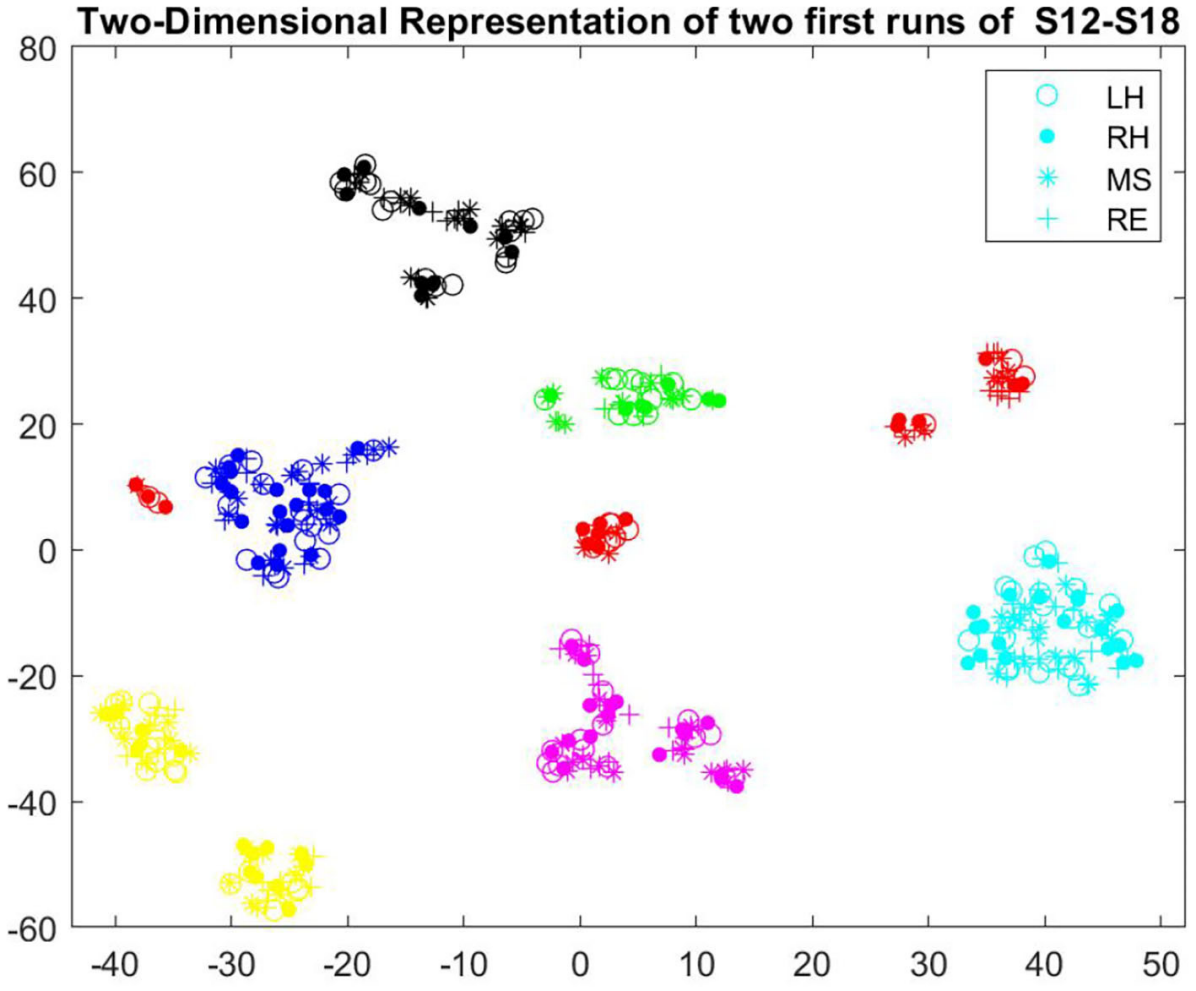
Two-dimensional representation of SCMs, projected by t-SNE, recorded in two first runs of sessions 12–18 (different sessions are illustrated in different colors, S12:green, S13: blue, S14: black, S15:magenta, S16: yellow, S17: red, S18: cyan).

**Reducing between-session variability:** From the beginning of the transfer phase, we started using a classifier trained over multiple runs from multiple sessions. However, we observed considerable shift between different sessions, resulting from different non-stationarity sources. Figure 5 illustrates the two dimensional representation of two first runs of sessions 12–18. All these sessions were 4-class training sessions (LEFT-HAND vs. RIGHT-HAND vs. MENTAL-SUBTRACTION vs. REST) recorded during the transfer phase. We used t-SNE (Maaten and Hinton, 2008) to project the data points (EEG covariance of one epoch/trial) in two-dimensional space. For dimensionality reduction using t-SNE to consider the non-linear geometry of the feature space, we used the Riemannian distance as the custom distance parameter. In addition, we set the effective number of local neighbors of each point (i.e., the Perplexity parameter) to the number of epochs extracted from each task in each run (i.e., 10).

As mentioned before, adaptive rebias FgMDM (Kumar et al., 2019) only addresses within-session variabilities, but not between-session ones. Thus, to reduce the latter we explored a new approach, which aimed at projecting all sessions (both training and testing ones) to a common reference. More precisely, we estimated a projector for each session, whose purpose was to project the Karcher mean of the 2-min baseline recorded at the beginning of that session (during which the pilot was in a resting with eyes open condition) to the common reference, i.e., the identity matrix. Then, all MT-BCI SCMs of that session were projected using this session-specific projector, as follows:

(11) $$\begin{array}{r} C_{i,j}^{\prime }={\bar{R_{j}}}^{-1/2}\times C_{i,j}\times {\bar{R_{j}}}^{-1/2} \end{array}$$

where $\bar{R_{j}}$ is the Karcher mean of epochs extracted from the baseline recorded at the start of each session, *j* denotes the session index and *C*_*i, j*_ is the *i*^*th*^ MT-BCI SCM sample of the *j*^*th*^ session. Such projected SCMs were then used as input to the adaptive rebias FgMDM classifier described above. The final classifier was thus trained on multiple sessions that were all projected to the same common reference, whereas the online data were also projected to this common reference, thanks to the projector built on the baseline of that session.

This approach was used for all sessions from Session 14, including during the day of the CYBATHLON competition. To the best of our knowledge, this new approach is the only one aimed at reducing between-session variability with Riemannian classifiers, in a completely unsupervised way, and without requiring MT-BCI data (but only a baseline). Moreover, such approach could also be used with non-Riemannian classifiers, e.g., with classical CSP-based BCIs, since CSP extracts features from covariance matrices as well .

It should be mentioned that, within the BCI community, it is still debated whether adaptive machine learning algorithms do favor user learning or not, and if so, how and how often the adaptation should be performed (see Scherer et al., 2018; Perdikis and Millan, 2020; Roc et al., 2020 for reviews). In this paper, we tried to favor a beneficial mutual adaptation between the user and the machine by using a machine learning adaptation that was purely unsupervised and class-unspecific. Indeed, our adaptive Riemannian classifiers only tracked and adapted to the global EEG changes, but not to the class-specific EEG ones. In particular, it should be stressed that our FgMDM classifier weights *W* (see Appendix A) were not changed by the adaptation—only the projections applied to the input covariance matrices (Equations 9 and 11) were adapted. This way we hoped to address global EEG non-stationarity while limiting the risk of an unstable feedback that would happen if constantly updating the classifier weights.

##### 2.4.2.3. Postprocessing

For the standard bar-feedback MT-BCI training (illustrated in Figure 4), the feedback was continuous and directly related to the classifier output. Here the FgMDM classifier outputs (Riemannian distances to class means) were transformed into pseudo-class probabilities *P*_*i*_, as $P_{i}=\frac{p_{i}}{\sum \left(p_{i}\right)}$, with $p_{i}=\frac{min_{i}d_{i}}{d_{i}}$, $d_{i}=d_{R}^{2}\left(C,\bar{C_{i}}\right)$, and *C* being the current EEG epoch covariance matrix (after projection and geodesic filtering). The feedback bar length was directly proportional to *P*_*i*_.

For the CYBATHLON game, the commands sent and the resulting feedback provided were not continuous but discrete, e.g., the car headlights were either on or off. Since sending erroneous commands was slowing the car, it was also important to reduce false positives, and thus to send commands to the game only when the BCI was confident enough that this was the correct command. Thus, for the CYBATHLON game, the BCI sent a command to the game only when the classifier identified the same class label consecutively for the last *N*_*e*_ EEG epochs, and with an output pseudo-probability greater than *P*_*c*_. In practice, we empirically determined suitable values for our pilot, and used (after a few trials and errors) *N*_*e*_ = 8, and *P*_*c*_ = 0.3 (for a 4-class problem, the minimum probability to select a class was thus 0.25). As a reminder, for the game we used sliding EEG epochs that were 1 s long, with 1/8 s step between consecutive epochs.

##### 2.4.2.4. Evaluation Criteria

To evaluate the user's progress, besides classification accuracy, we also used studied two additional metrics that study users' EEG changes: *classDis* and *Test-Train Adaptation*.

*classDis* measures how distinct and stable the EEG patterns produced by the user are, independently of any classifier (Lotte and Jeunet, 2018). For a two-class problem, *classDis* is defined as follows:

(12) $$\begin{array}{r} classDis\left(c_{i},c_{j}\right)=\frac{d_{R}\left({\bar{C}}^{\left(c_{i}\right)},{\bar{C}}^{\left(c_{j}\right)}\right)}{\frac{1}{2}\left(\sigma_{c_{i}}+\sigma_{c_{j}}\right)} \end{array}$$

where the numerator computes the distance between class means and the denominator is the summation of average squared distances around the Karcher mean of each class. For multi-class problems, the numerator of *classDis* computes the distance of the Karcher mean of each class ${\bar{C}}^{\left(c_{i}\right)}$ from the Karcher mean of all the data $\bar{C}$, as follows:

(13) $$\begin{array}{r} classDis\left(c_{i}\right)=\frac{\underset{i}{\sum }d_{R}\left({\bar{C}}^{\left(c_{i}\right)},\bar{C}\right)}{\underset{i}{\sum }\sigma_{c_{i}}} \end{array}$$

where ${\bar{C}}^{\left(c_{i}\right)}$ and $\bar{C}$ are the Karcher mean of each class and of all the data, respectively. Overall, *classDis* could thus measure whether the user's EEG patterns for each class become increasingly more distinct with training, independently of any classifier.

However, *classDis* may not capture all forms of BCI user learning. In particular, since *classDis* is classifier-independent, it may not capture some potential user adaptation to the BCI system and classifier. Indeed, with BCI training, some users may learn to produce EEG patterns that become increasingly more systematically similar to those expected by the classifier, i.e., to those used to train the classifier. In turn, this would lead these EEG patterns to be increasingly more correctly recognized, and hence the user to reach increasingly better MT-BCI control. Thus, in order to evaluate whether some user learning occurred as users' EEG signals changing to adapt to the BCI system/classifier, we need a new metric. In particular, we need to quantify how much the users' online EEG data, i.e., test data distribution, becomes similar to the EEG data used to train the classifier, i.e., training data distribution. We propose here a new criterion in the Riemannian framework of covariance matrices to do so.

To estimate the similarity/dissimilarity between training and test sets, we chose multiple landmarks from the training and test sets: we used the Riemannian means of each class as well as the overall Riemannian mean of all samples (all classes together). The smaller the distances between these landmarks in the training and test set, the more similar their distributions. Here, the distance between these landmarks between the training and test sets are normalized by either the within-class variance or the overall variance in the training set. The average of these normalized distances represent the similarity between the test and training sets, and can thus be used to measure Test-Train Adaptation (TTA):

(14) $$\begin{array}{l} TTA=\frac{1}{\left(N_{c}+1\right)}(\sum_{i=1}^{i=N_{c}}d_{R}({\bar{C}}_{train}^{\left(c_{i}\right)},{\bar{C}}_{test}^{\left(c_{i}\right)})/\sigma_{train}^{\left(c_{i}\right)}\\ \;+\;d_{R}({\bar{C}}_{train},{\bar{C}}_{test})/\sigma_{train}) \end{array}$$

where ${\bar{C}}_{train}^{\left(c_{i}\right)}$ and ${\bar{C}}_{test}^{\left(c_{i}\right)}$ denote the Riemannian mean of class *c*_*i*_ in training and test sets, respectively. $\sigma_{train}^{\left(c_{i}\right)}$ denotes the standard deviation of class *c*_*i*_ in training set and ${\bar{C}}_{train}$ denotes the global mean of all training samples. Here, all covariance matrices were recentered using Equations (9) and (11), as done online to reduce between- and within-session non-stationarities. *N*_*c*_ is the number of classes. This TTA metrics displays some similarities with the “classifier precision” metrics from Perdikis et al. (2016). Indeed, this latter metrics quantifies the similarity between test trials and the feature class distribution of a classifier. It was used in Perdikis et al. (2016) to assess how much an adaptive classifier class distribution could adapt to online testing EEG data. Here we use TTA in the opposite way, to assess how much the user's testing EEG data adapt to the classifier class distribution. Overall, a decreasing *TTA* metric over online sessions would suggest more adaptation of test sets to the training set. In other words, this would mean that the user is producing EEG patterns increasingly more similar to what the classifier expects for each class.

### 2.5. Neurophysiological Analysis

For all our neurophysiological analyses, we rejected artifacted epochs (i.e., artifacted trials) from our data, i.e., epochs with absolute amplitude higher than 70 μV. Neurophysiological analyses were made for the 2-class BCI training phase and the transfer phase (i.e., 2-class online BCI and 4-class online BCI). We used a robust variant of the Fisher score (FS) to evaluate the discriminability between the different classes for different frequency bands and brain areas:

(15) $$\begin{array}{r} FS=\frac{\overset{N}{\underset{i=1}{\sum }}{\left(m_{i}-m_{0}\right)}^{2}}{\overset{N}{\underset{i=1}{\sum }}mad_{i}^{2}} \end{array}$$

where $m_{0}=\overset{n}{\underset{i=1}{\sum }}\left(m_{i}\right)$, *m*_*i*_ is the medians and *mad*_*i*_ the Median Absolute Deviations (MAD) of the EEG signal power spectral density distributions for the *i*^*th*^ mental class in a specific frequency band and channel, within each run. N is the number of classes. This FS formulation uses a median and a MAD instead of a mean and a standard deviation in the usual FS, the former estimators being more robust estimators than the later ones, for the expected value and dispersion around the expected value, respectively.

In our analyses, we considered three different frequency bands: the α-band (8–12 Hz), the low β-band (13–20 Hz) and the high β-band (21–30 Hz), since all these bands are known to be involved in cognitive and motor imagery tasks (Friedrich et al., 2013). We chose not to analyse the sessions of the exploratory phase since both the setup and the user training protocol were not fixed yet.

**Figure 6:**
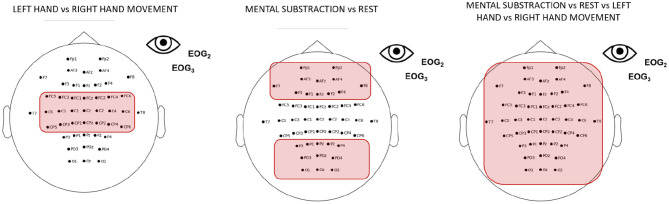
Selected electrodes for neurophysiological analysis. From **left** to **right**: Electrodes of the motor area for the LEFT- vs. RIGHT-HAND motor imagery tasks, electrodes of the frontal, parietal and occipital areas for the MENTAL SUBTRACTION vs. REST mental tasks and all the electrodes for the 4-class BCI.

During the second user training phase, the pilot was trained with two pairs of tasks, including training to control a 2-class *motor imagery* BCI (imagination of a left vs. right hand movement). Thus, we computed the mean of the FSs obtained over the electrodes of the motor area (Pfurtscheller and Neuper, 2001) (see Figure 6) for each frequency band to evaluate in which frequency band the discriminability between the 2 classes was the highest. During this second phase, the pilot was also trained to control a 2-class BCI with *cognitive* tasks (REST state vs. MENTAL SUBTRACTION). As for the motor imagery tasks, we computed the mean of the FSs obtained for each frequency band. This time, we selected electrodes of the frontal, parietal and occipital areas (see Figure 6). We chose to focus on those specific areas because MENTAL SUBTRACTION can involve frontal as well as parietal processes (Chochon et al., 1999).

Finally, during the transfer phase in which it was a 4-class *mental tasks* BCI, we did the same analysis with all the electrodes (see Figure 6).

We assessed possible learning effects by computing the Pearson correlation between the FS and the run index, as in e.g., Perdikis et al., 2018, for each frequency band and each phase of the training.

## 3. Results

### 3.1. Behavioral Results

**Figure 7:**
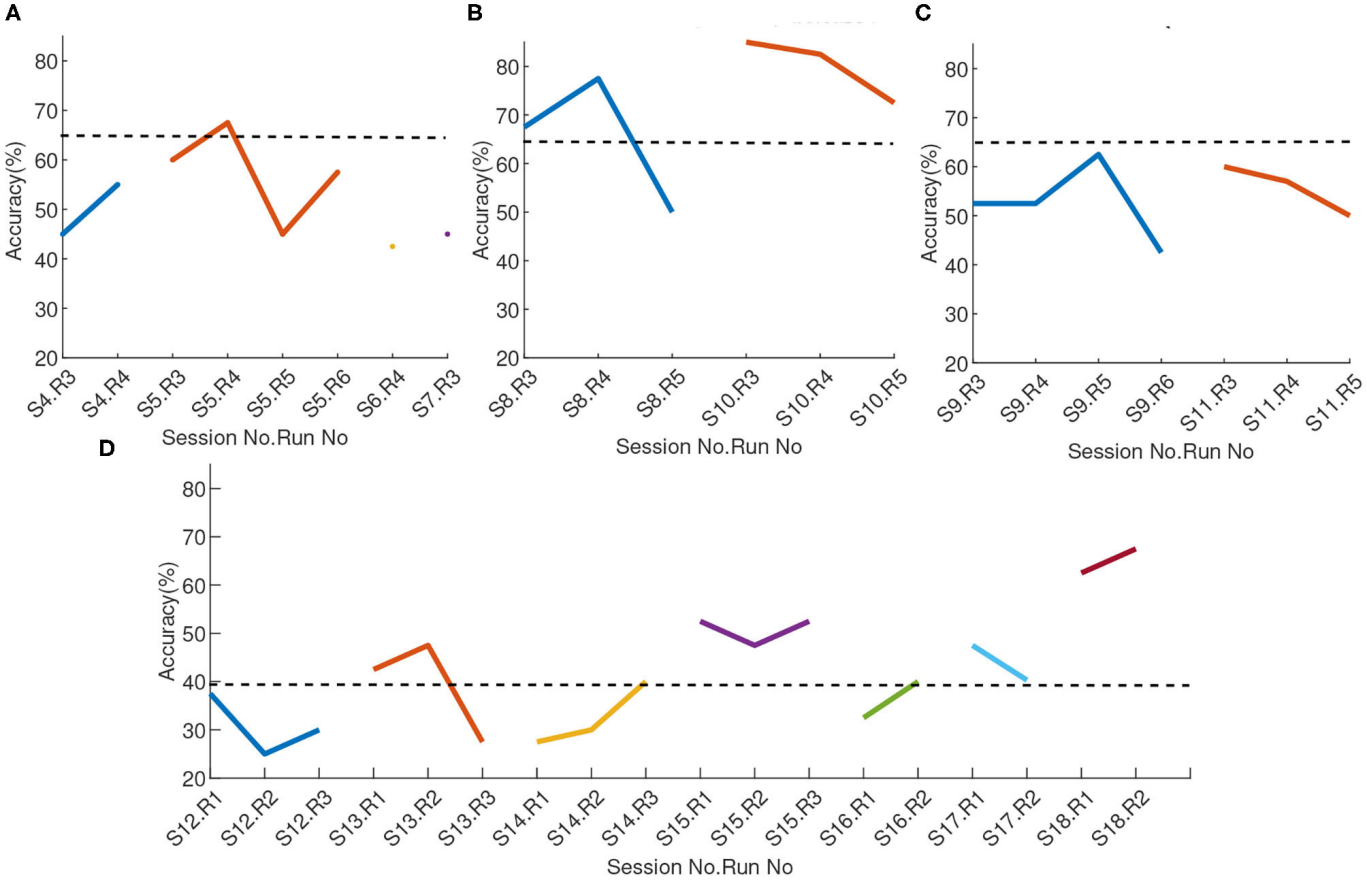
Online classification accuracy across sessions and runs **(A)** Exploratory phase, **(B)** MENTAL-SUBTRACTION vs. REST of two-class training phase, **(C)** LEFT-HAND vs. RIGHT-HAND of two-class training phase, and **(D)** Transfer phase. Each point represent the accuracy of a run, different colors represent different sessions. The solid lines show the online accuracy and the dashed lines show the upper confidence limit of the chance-level at α = 0.05, according to Müller-Putz et al. (2008).

**Figure 8:**
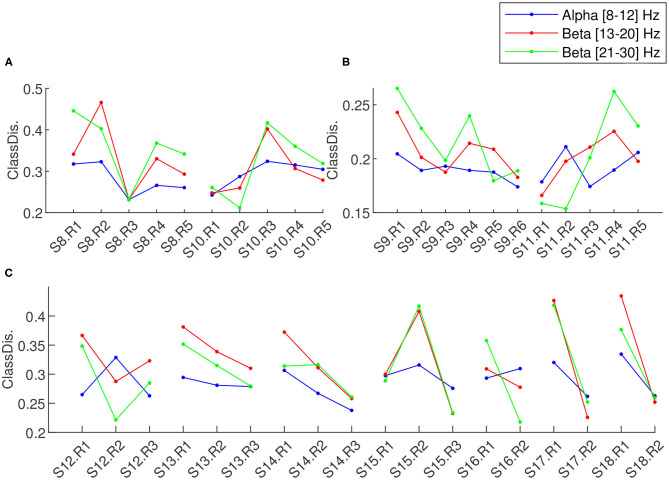
Class discrimination for each run of each session of **(A)** MENTAL-SUBTRACTION vs. REST of two-class training phase, **(B)** LEFT-HAND vs. RIGHT-HAND of two-class training, and **(C)** Transfer phase. Different colors represent different sessions, each point represent a run.

To evaluate the learning progress across different sessions and within each session, we studied both the BCI performance in terms of online classification accuracy (Figure 7) and offline *classDis* (Figure 8) for feedback training sessions. We computed *classDis* for all runs, including the acquisition runs (for sessions that had such runs) and the test runs (Figure 8). For game performance we evaluated the user performance in terms of required time to complete the game.

#### 3.1.1. Exploratory Phase

Due to the exploratory and non-systematic nature of this phase, online classification accuracies were not recorded for every run or session. The mean online accuracy for the runs where this data was recorded is reported in Figure 7A. The dashed line in this figure shows the upper-confidence limit of the chance level for α = 0.05 and 20 trials/class (Müller-Putz et al., 2008). The non-systematic ways the sessions of that phase were performed also made comparing *classDis* across sessions non-meaningful. We thus did not report it for that phase.

#### 3.1.2. 2-class Training Phase

For the two-class training phase, two different pairs of tasks were used, with different discriminablity. Thus, we report the results related to MENTAL-SUBTRACTION vs. REST tasks imaginations (Session 8 and 10) in a sub-plot (B) and the results related to session 9 and 11 (LEFT-HAND vs. RIGHT-HAND motor imagery experiments) in sub-plot (C) in Figures 7, 8. For the 2-class training phase, the upper confidence limit of the chance-level for classification accuracy was computed for a significance level of α = 0.05 and for 20 trials/class (Müller-Putz et al., 2008). We can observe that the performance with MENTAL-SUBTRACTION vs. REST is clearly superior to that with LEFT-HAND vs. RIGHT-HAND. The learning progress in terms of the Pearson correlation of the run classDis with the run index in different frequency sub-bands did not reveal a significant learning for any band nor any class pair.

#### 3.1.3. Transfer Phase

The sessions of the transfer phase include both 4-class feedback training and game training. The results of the feedback runs in terms of classification accuracy and *classDis* are reported in sub-plots (D) and (C) of Figures 7, 8, respectively. In Figure 7D, the upper confidence limit of a random classification accuracy in the transfer phase, illustrated using a dashed line, was computed for a significance level of α = 0.05 and for 10 trials/class (Müller-Putz et al., 2008). In terms of classification accuracy, a very clear performance improvement between sessions can be observed. Indeed, performances for the 4-class BCI started at near chance level (between 25% to 37.5% for the first 4-class session) and finished at 67.8% for the last run of the last session, while generally increasing (with fluctuations) in between. A Pearson correlation between classification accuracy and run index revealed that this learning effect is significant (ρ = 0.65, *p* < 0.01). Comparing 4-class feedback training classification accuracy before (Sessions 12 and 13) and after using the proposed adaptive approach (Sessions 14–18) provides interesting insights. In terms of run accuracy, a t-test revealed a trend toward run accuracy being higher with the adaptive approaches rather than without (*p* = 0.07). More interestingly, we also estimated the learning progress using the Pearson correlation of the run accuracy with the run index. Such analyses revealed no learning effect before using the adaptive approach (ρ = 0.18, *p* = 0.73), but a clear learning effect after the user started to use the adaptive approach (ρ = 0.68, *p* = 0.015), with a mean accuracy of 44.5% for the two first sessions and 54.45% for the last two sessions. This thus suggests that our proposed adaptive approach may have contributed to improve user training, by providing a more stable classifier, and therefore a more stable feedback.

On the other hand, offline *classDis* did not show any significant correlation with run index in any band.

**Figure 9:**
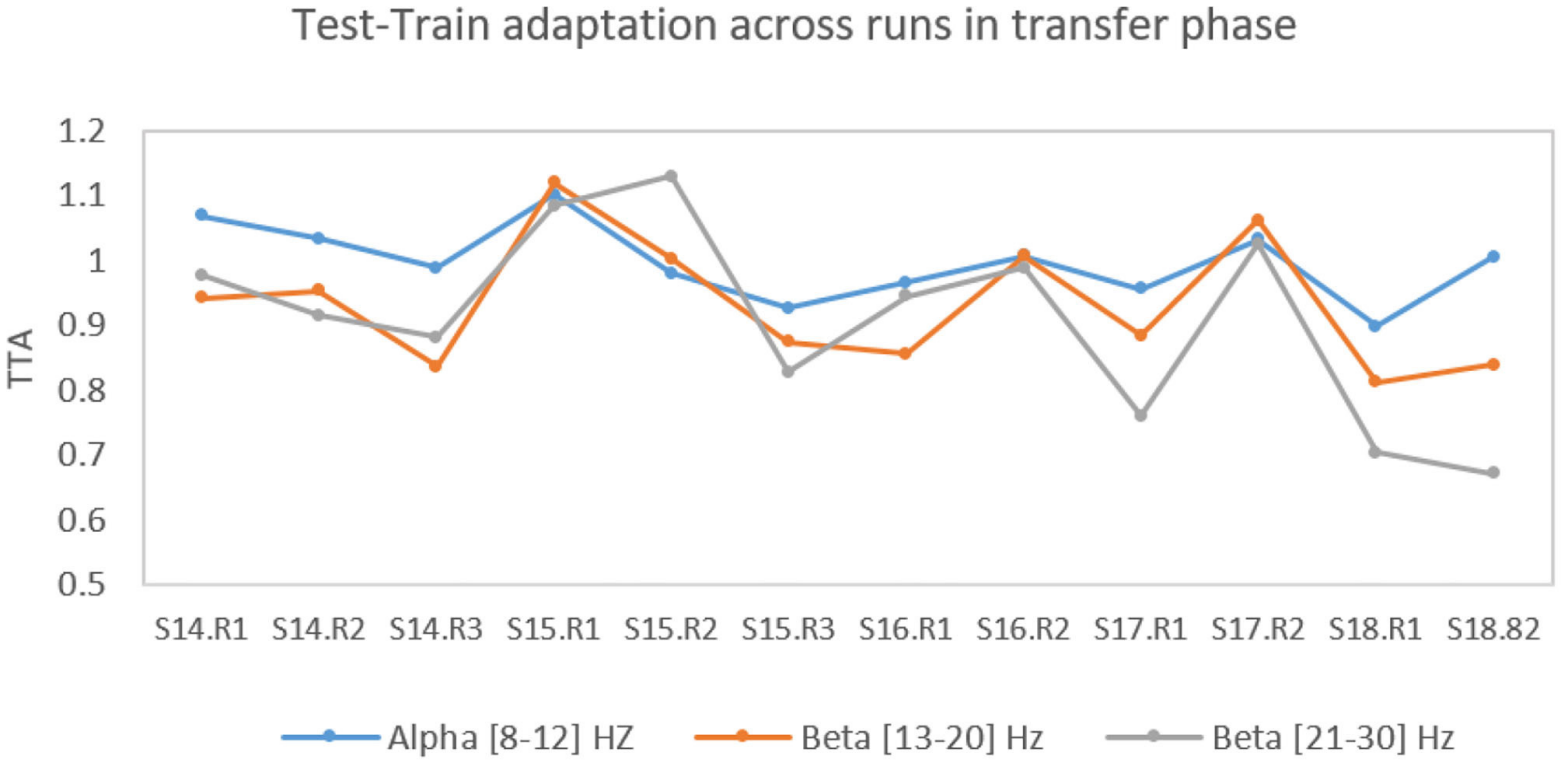
Evolution of the proposed Test-Train Adaptation (TTA) metric over the runs in the transfer phase in different frequency bands.

This apparent inconsistency between significant improvement of online classification accuracy but non-significant improvement of *classDis* in the transfer phase, could be explained by studying our new metric *TTA* in the different frequency bands across the different runs from session S14–S18, see Figure 9.

Here the (offline) training test was the data from Sessions S8-S13, and the online test set Sessions S14-S18. Indeed, the FgMDM classifier weights were optimized on this training set and kept fixed for this online test set. Pearson correlation between *TTA* and run index confirmed a significant negative correlation in the high β-band (ρ = −0.57, *p* = 0.05) with a mean TTA value of 1.00 for the first two sessions and of 0.79 for the last two sessions. In α and low β-band the correlations of TTA and run index were not significant though (ρ = −0.44, *p* = 0.14);(ρ = −0.26, *p* = 0.40). Overall, this suggested that our pilot learned to increase his BCI classification accuracy not by producing increasingly more distinct EEG patterns (as measured by *classDis*), but by learning to produce EEG patterns that increasingly better matched those expected by the classifier for each class (as measured by TTA).

**Figure 10:**
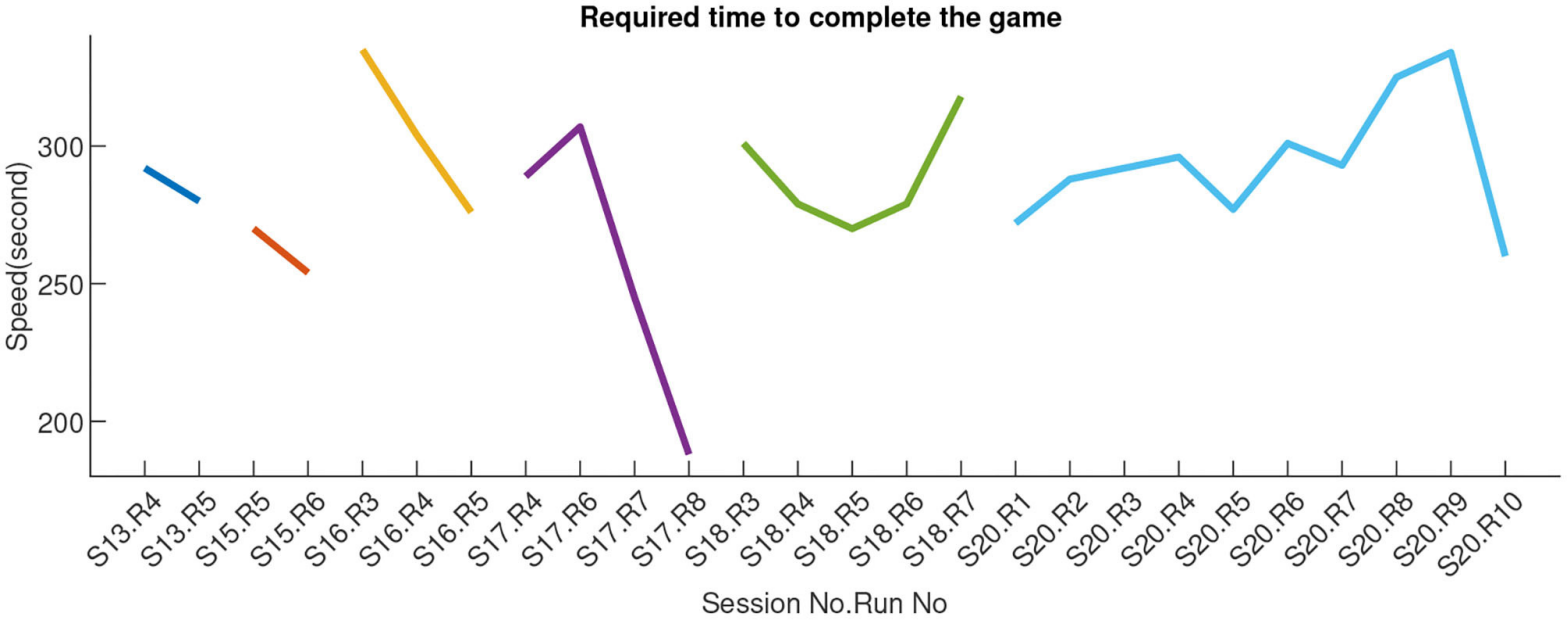
Game performance in terms of required time for completing the race. Different colors represent different sessions. Session 19 game times were not recorded.

Game performance over the runs of each session, in terms of the required time to complete the game (in seconds), is illustrated in Figure 10.

For at least 4 of the 7 game sessions, we observed improvement in performance across runs, within-sessions. However, between-sessions, there does not seem to be any learning effect with improvement across sessions. Some sessions/runs were at times much better (notably session 17), but such performance was not sustained in subsequent sessions. This lack of apparent learning to control the game better was confirmed by the lack of significant correlation between game completion time and run index (ρ = 0.17, *p* = 0.41).

### 3.2. Competition Results

Six international BCI teams participated in the 2019 CYBATHLON BCI competition in Graz during which the race completion time was the winning criterion. The maximum duration of the race was 4 min. If two (or more) pilots could not finish the race within the allotted time, the distance traveled was used to arbitrate the pilots. Victory was played in three rounds. Two qualifications rounds to select the three best pilots and a third round to determine the final ranking. Our pilot did not complete the races of the first two rounds in less than 4 min and therefore was not qualified for the track A finale (i.e., the finale with the best pilots) but the track B finale. During this finale, he did not finish the race in the allotted time (distance traveled: 399.8/500) and was ranked 5th out of 6. It should be mentioned that we faced technical issues during the competition. Firstly, instead of only two, the pilots had to go through four qualification races due to communication problems between the racing team and the organization's material which affected several pilots in the first two races. Secondly, we had to change an EEG electrode on stage, between two races, as it was not working anymore.

### 3.3. Neurophysiological Results

#### 3.3.1. Exploratory Phase

As mentioned before, the exploratory phase was more of an adaptation phase for both the pilot and the experimenters rather than a training phase. We used those 7 sessions to adapt the cap, the algorithms and for the pilot to be more familiar with the system. Thus, we did not do any neurophysiological analysis on that phase.

#### 3.3.2. 2-class Training Phase

**Figure 11:**
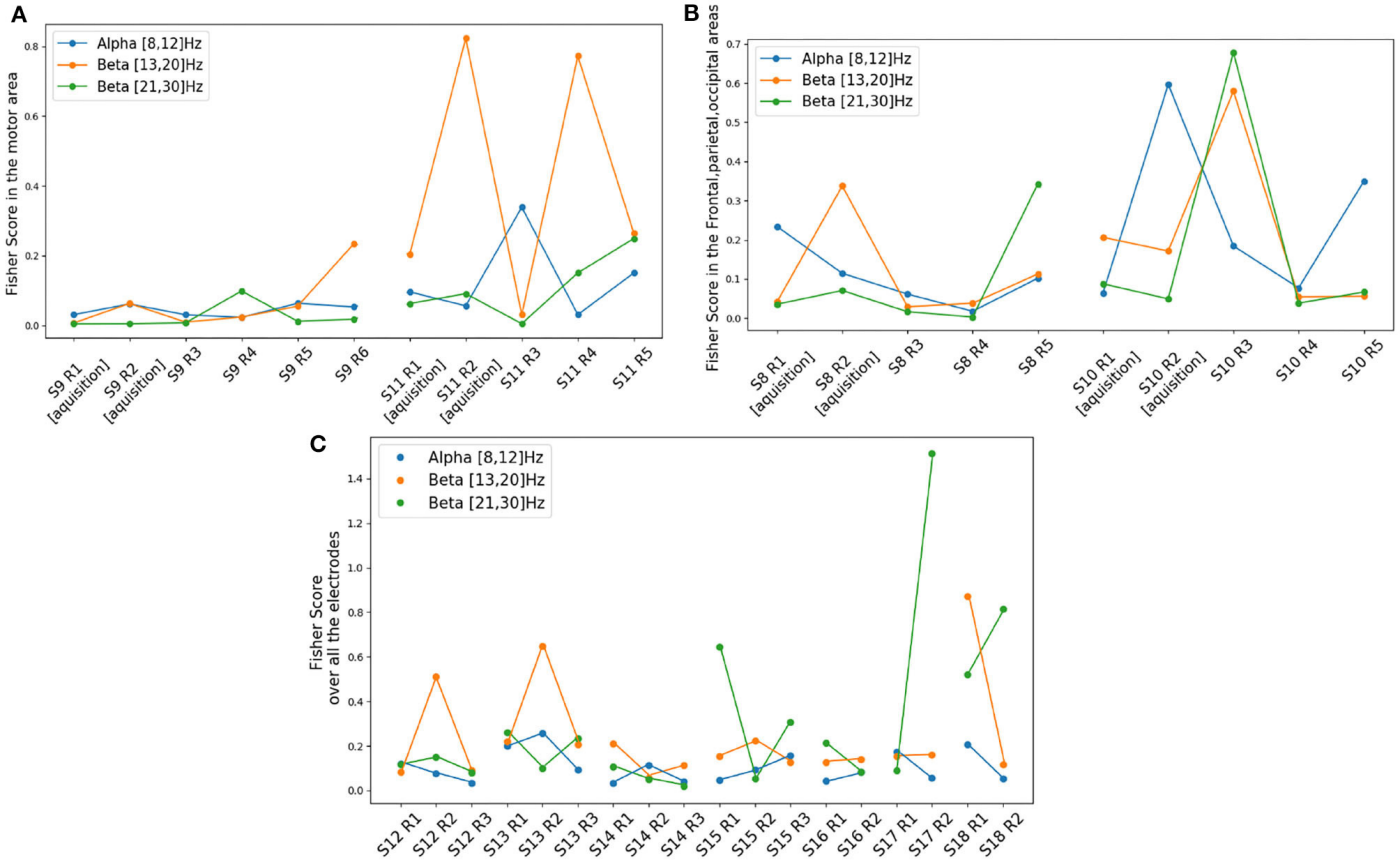
Mean Discriminability score (Fisher Score) for each frequency band for different conditions across the sessions. In **(A)**: Mean FS in the motor area (2-class motor imagery BCI) for three different frequency bands. In **(B)**: Mean FS in the frontal, parietal and occipital areas (2-class mental task BCI, i.e., REST vs. MENTAL SUBTRACTION) for three different frequency bands. In **(C)**: Mean FS in all the electrodes (4-class BCI), for the three different frequency bands.

During the 2-class training phase, we trained our pilot for four sessions in 2 weeks (i.e., sessions twice a week). Two sessions were dedicated to motor imagery BCI (LEFT- vs. RIGHT-HAND movement imagination) and two to non-motor mental-task BCI (MENTAL SUBTRACTION vs. REST). During the 2-class motor imagery BCI (Figures 11A,B), we observed a learning effect in the low β-band (13–20 Hz in orange). Indeed, there is a significant correlation between run indexes and the FS in this frequency band (ρ=0.70, *p* < 0.05). No other significant correlation were observed.

#### 3.3.3. Transfer Phase

During the transfer phase in which we alternated between the game and a more classic 4-class BCI during 7 sessions, We can observe in Figure 11C that the mean discriminability over all 46 electrodes seems to be increasing over time in both low and high β-bands (13–20 and 21–30 Hz). However we did not observe any significant correlation between FS and the run number. Such results seem in line with the results obtained with *classDis* and *TTA* previously: our pilot did not seem to increase the discriminability of his EEG patterns with learning (as measured with FS or *classDis*), but rather to produce EEG signals increasingly more systematically similar those used to train the classifier (as reflected by *TTA*).

**Figure 12:**
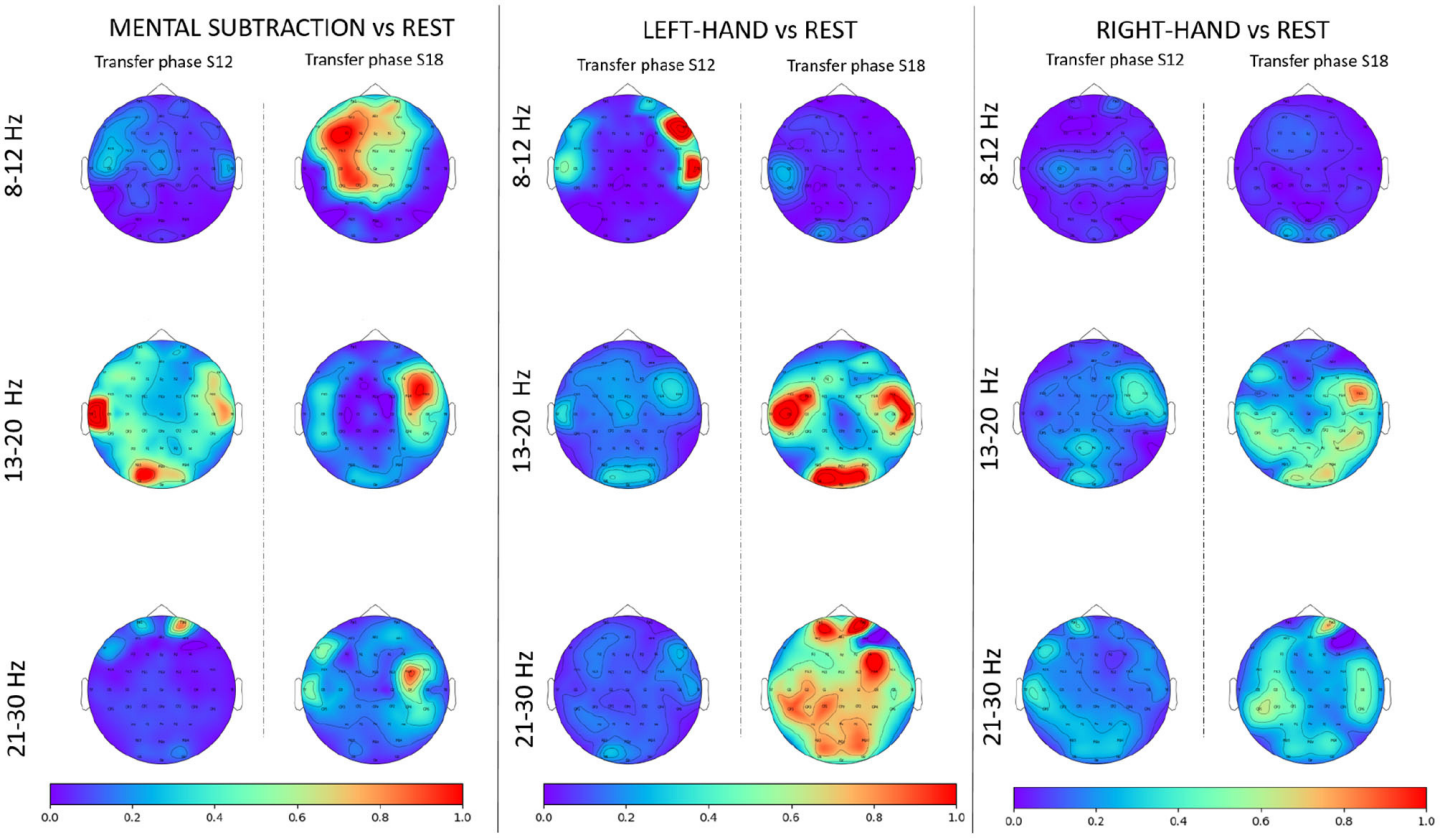
Topographic maps (Fisher Score) for each frequency band for the 3 active classes against rest state. For each condition, we compared the mean FS over the runs of the first session of the transfer phase and the one of the last session of the same phase.

In order to verify the soundness and relevance of the subject's class-wise EEG patterns, we used topographic maps of the Fisher Score for the 3 active classes against the rest class, during the transfer phase. We did so for each frequency band and compared the first session (i.e., mean of the FS over all runs) of this phase with the last one (Figure 12). Results showed that there was a learning effect in the expected brain areas from a neurophysiological point of view: FS increased in the frontal area for MENTAL SUBTRACTION vs. REST. We also observed that, when comparing motor tasks with REST, there was an increase in FS in the motor areas (around C3 and C4), as well as in visual areas. However, we did not observe any lateralized pattern, but rather a bilateral one. This would explain why our pilot had difficulties to produce distinct EEG patterns for the two hand motor imagery tasks.

### 3.4. User Experience

#### 3.4.1. Questionnaires

**Figure 13:**
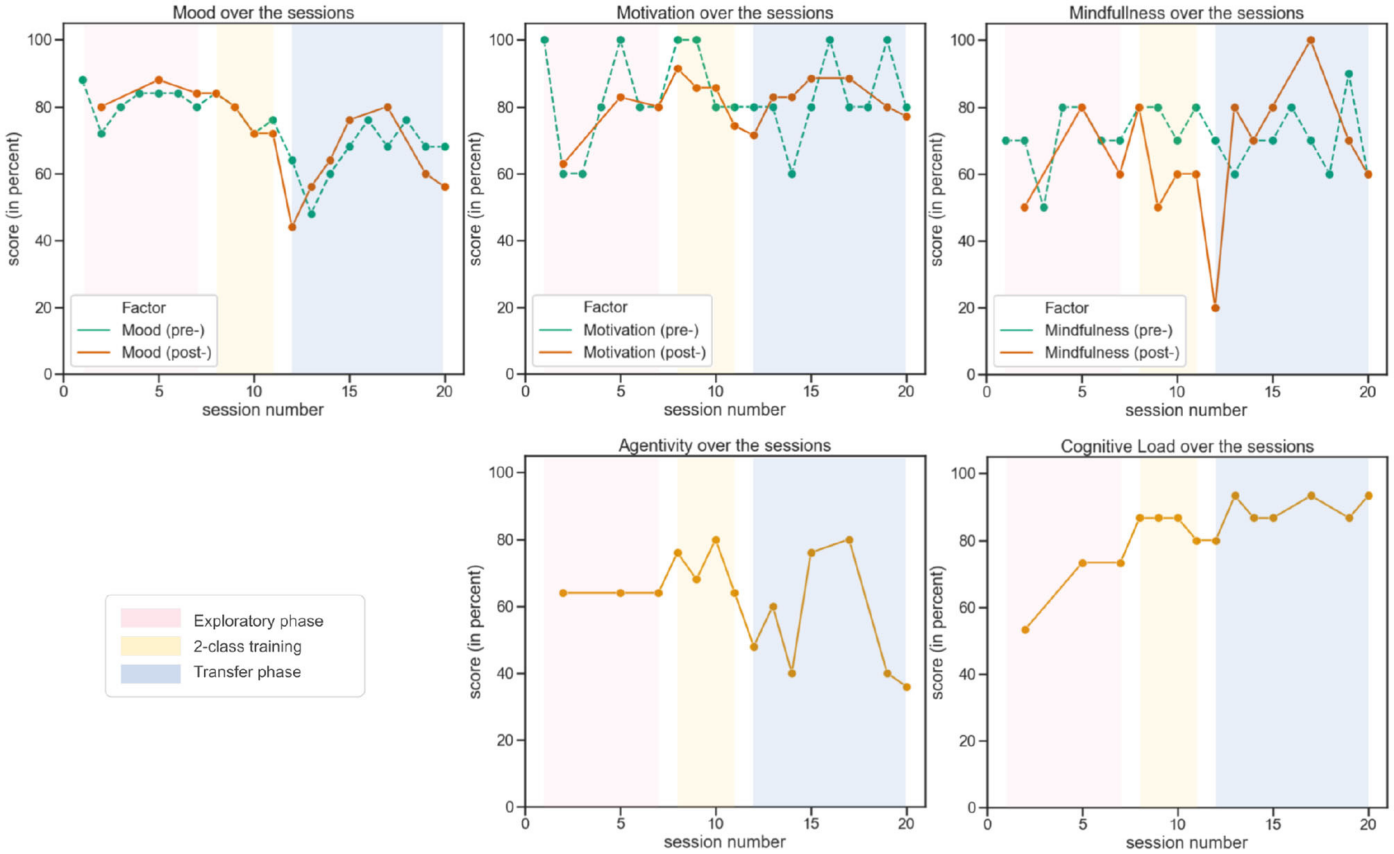
Evolution over sessions of user experience metrics, i.e., user mood, motivation, mindfulness, agency, and cognitive load.

The questionnaires described in section 2.3.2 provide us with scores for 5 factors, i.e., mood, motivation, mindfulness, agency, and cognitive load (the last two being assessed post session only). For the sessions in which the questionnaires were administered, the results are reported in Figure 13.

For the pilot, the first session of the transfer phase (12) was particularly difficult. He encountered a rather low online classification performance and reported significant fatigue. As shown in Figure 13, this resulted in a particularly low mindfulness at the end of this session (12), as well as a rather low mood both at the end of this session and at the beginning of the next one (13). Regarding motivation, although quite variable from one session to the next, remained overall rather high throughout training as reflected by the interview. Also, a high level of agency seems to be associated with high peak times when playing the game (cf. Figure 10).

Finally, during the course of the training, the cognitive load gradually increased and was at its highest during the transfer phase (4-class user training and game).

#### 3.4.2. Interview

A semi-structured interview of the pilot was conducted after the competition in order to assess different dimensions of user experience including acceptability, acceptance and satisfaction. The pilot's answers provided herein-after are organized based on the four main steps of the “adventure,” i.e., (i) before the BCI training, (ii) during the BCI training, (iii) during the competition and (iv) after the competition.

During the interview, the pilot indicated that he had never used a BCI before. When he was offered to take part in the CYBATHLON, he first felt curious and considered this opportunity as challenging and “awesome.” He was extremely attracted by the idea of integrating a research project, to contribute to the emergence of a technology, from the very beginning of the process. Being part of this new “adventure,” culminating with the CYBATHLON competition, was his main motivation. He also mentioned that it was very easy for him to envision many promising applications of BCIs for the general public. However, he did not mention having any expectation related to the improvement of his clinical condition. Rather, he was excited to be part of a team, represent his country in an international competition, meet with new people and discover a field of which he knew nothing before. The pilot's close family and friends also strongly supported him in his project. Thus, the pilot's BCI acceptability a priori was high. He was very motivated, with a high perceived usefulness of the technology and very positive representations. Moreover, He will mention later in the interview that he did not expect the training to be so hard, showing that he also perceived BCIs as easy to use. This high acceptability resulted in a great engagement of the pilot in the training procedure, which is reflected by the results of the questionnaires depicted in section 3.4.1.

During the second phase of the interview, dedicated to the BCI training phase, the pilot mentioned that keeping in mind the final objective, namely the competition in Graz, enabled him to keep his motivation high all along the training program. Nonetheless, it was not easy as he had under-estimated the mental effort required for completing BCI sessions. This resulted in huge tiredness after some sessions. He also found it difficult to manage, from a psychological standpoint, the between-session variability in terms of performances as he felt like he had no control over it. This second difficulty led to high frustration levels during and after some of the sessions that he has had to learn to master. The pilot mentioned that beyond the objective of the competition and the trip to Austria with his friends, the emotional as well as the cognitive support provided by the team members, who were much present and explained the potential origins of variability in performance, helped him to remain motivated.

As the CYBATHLON competition approached, the pilot reported feeling both extremely excited, impatient and a bit stressed. He also felt the stress of the team, especially when technical issues occurred on stage. During the race, the pilot switched between frustration and relief periods. But in the end he was mainly proud and happy. Most importantly, he insisted on the fact that he loved the way the competition was organized. It was a real show, with an over-excited audience and interviews. He felt like “any other athlete, on equal terms.” He adds, “I was not a disabled person helped by researchers. I was the pilot of a team.” the pilot qualified this feeling as “refreshing” and wished that this kind of event where disability is not associated with pathos and sentimentality were more frequent.

Now that the “Adventure” is over, the main feelings of the pilot when he recalls the whole process are a huge satisfaction and happiness. It was a bit hard at the beginning not to have the training routine anymore. Now, he would like to compete again and increase his performance. He thinks that mental coaching and meditation sessions could help. Beyond the CYBATHLON experience, the pilot affirmed that he would recommend non-invasive BCIs to anyone who is “ready to train hard.” He also insisted on the importance to explain first how this (impressive) technology works in order to play down the assumed danger/difficulties associated with these technologies. He also mentioned that he would ready himself to train hard to learn to use them for controlling assistive devices in the future. He is certain that non-invasive BCIs will be made accessible and reliable for home automation notably in the near future. This shows that the pilot's experience with BCIs resulted in a high acceptance, associated with a high perceived usefulness and a certain confidence in the fact that these technologies will soon be reliable and easy enough to be usable and useful outside of laboratories.

## 4. Discussion

During this study that lasted about 3 months, we conducted multiple BCI experiments with a tetraplegic user both inside and outside the laboratory. This encouraged us to tackle multiple challenges associated with BCI use over multiple days, in “real life,” with an actual end-user. Challenges were related to non-stationarity problems, user training but also managing the short time we had before the competition.

At the beginning of the transfer phase (Sessions 12 and 13), even when using a classifier trained on multiple runs and sessions, the resulting classification accuracy still suffered from a high variability between runs and sessions. Using a newly proposed adaptive approach to model both within and between-session variabilities, from Session 14 onwards, led to improvement in classification accuracy across runs and sessions. This observation confirmed the effectiveness of the proposed adaptive Riemannian classification approaches for addressing non-stationarity effects. During the training, we observed user learning across sessions, notably in the transfer phase, and particularly when using the adaptive approach with the bar feedback. However, we did not observe any clear increase in game performance across sessions for controlling the CYBATHLON BCI game. We only observed within-session game performance improvement, but such improvement did not sustain to the subsequent sessions. Within-session game improvement was probably due to the within-session adaption of our classifier, which was progressively adapting the classifier to the game context. Indeed the classifier was trained on the bar-feedback context, and not the game context. Indeed, the asynchronous nature of the game prevented us from using it to collected ground truth EEG data to calibrate the BCI. However, the differences between the standard bar-BCI training and the CYBATHLON game training, e.g., the continuous vs. discrete feedback, the positive-only vs. positive and negative feedback or the simple vs. complex visuals would likely lead to change in EEG patterns. Overall, this thus confirmed results from Perdikis et al. (2018) which stated that both standard feedback BCI training and application game training were different tasks, that both needed a dedicated training, possibly both for the user and for the machine (classifier).

In order to avoid impeding the user learning, it thus seems necessary to provide an accurate and stable feedback. In our study, unsupervised adaptive methods—that model global EEG changes within and between sessions, seemed useful to improve feedback stability and BCI performances. This proposed adaptive approach only models and corrects global EEG distribution shifts within (using adaptive rebias FgMDM) and between sessions (using between-session baseline projection) but not task-related EEG changes. This way, we hope it only contributed to stabilizing BCI feedback, but not in making the user “lazy,” by enabling him to overly rely on the ongoing machine learning doing all the work, as observed in Perdikis et al. (2016). Therefore, using the proposed adaptive approaches, which reduce the shift within and between sessions, we probably reduced the risk of confusing the user with a continually changing feedback.

Interestingly enough, the statistically significant improvement that we observed in both online classification accuracy and user adaptation to the BCI classifier training set (TTA metric), together with the non-significant improvement in *classDis* provide some evidence for a relatively new type of user learning. Indeed, our user learned to adapt his EEG signals to the BCI classifier, instead of increasing his EEG separability between classes (the typically studied type of BCI user learning). This potentially open doors to new ways to assess and study BCI user learning in the future. Naturally, this result is here shown for a single subject, and would thus need to be further studied with several other subjects, and other BCI designs, e.g., with non-adaptive classifiers.

As shown in Figure 13, the pilot's cognitive load showed an increasing trend throughout the training. At first, this may seem surprising since task execution supposedly requires less load as expertise increases. Throughout progressive training, one might thus have expected a more stable or even decreasing load: the training could build and automatize patterns associated with MTs (Sweller et al., 2019) thereby freeing up processing resources for the execution and learning of new aspects of training exercises, e.g., for controlling the game. We can first hypothesize that the decrease in the load relative to increased expertise was lower than the increase associated with the introduction of more complex and demanding exercises. Another line of interpretation would be to consider the causal factors of the cognitive load related to the environment. For example, stress, emotions and uncertainty can limit working memory capabilities (Choi et al., 2014) and thus increase cognitive load and impede learning. An acceleration of the training schedule (more sessions per week as the competition was growing nearer) and the approach of the competition might have triggered this type of process. It might therefore be recommended to carefully assess these elements in the future and to develop methods to support the user and help them to reduce the high load resulting from stress, emotions or uncertainty.

## 5. Lessons Learned

The CYBATHLON BCI series 2019 was, for the authors of the paper, a wonderful opportunity to experiment a long term BCI training with a end-user and a clear final objective and deadline: the CYBATHLON BCI competition. Yet, it was a first for our team and we learned a lot from our mistakes.

First, due to the short schedule, decisions had to be taken quickly without being able to step back. We believe that we took final decisions quite fast that were not the most relevant ones. One of those decisions was the tasks the pilot had to perform to control the BCI and more specifically the motor imagery tasks. Indeed, as our pilot was tetraplegic since 2007, it was not easy for him to do those tasks but also to generate distinct brain signals for those two tasks (i.e., LEFT- and RIGHT-HAND movement imagination). Spending more time on screening could have help us choosing tasks he was more comfortable with but also that were easier to discriminate.

Another challenge we had to face was the design and implementation of new algorithms while training our pilot such as using Riemannian geometry online and addressing the issue of non-stationarity effects. It was, at times, confusing and stressful for both the pilot and experimenters to train and test at the same time.

Regarding our adaptive approaches, for the game, the number of trials for the different classes was not equal, with the NOINPUT class (rest) being over represented (once a command was issued, the pilot could rest). This could have biased our within-session adaptive approach toward that class. We need to address this issue in the future.

Moreover, the pilot's testimony regarding the support of the team highlights three very important aspects that should not be neglected. Indeed, it seems necessary that researchers/clinicians clearly introduce and discuss with the user/patient in advance the system, the way it works, the level of performances that can be expected, the duration and potential difficulty of the training, etc. It is also of utmost importance to provide a comforting presence all along the training phase. Finally, defining clear objectives will most likely favor the engagement of the user/patient, provided that the two previous recommendations of cognitive and emotional support are followed.

Overall, the whole training protocol included 20 training sessions, arranged in three phases. This was mostly due to its exploratory nature. If we were to do it again, we would make the exploratory phase much shorter than the 7 sessions performed here. We would focus on screening various MTs in a systematic way to identify faster and better the ones to further train in the subsequent phase. The 2-class training phase appeared as useful to train our pilot in a progressive way. Indeed, an initial test with all 4-classes at once quickly overwhelmed our pilot. He then appreciated training first with pairs of MTs only. Finally, for the transfer phase, ideally, we should make that phase longer—to train the pilot more, and include game training earlier in that phase.

Finally, the environmental conditions during the competition were far from the ones we had at the pilot's home or in the lab. Indeed, during the training we were making sure that there was no disturbing element such as noise or movement in front of the pilot. In contrast, during the competition, the public was supporting pilots and therefore moving and making noise. In addition to the stress due to the competition, we were far from the training conditions. In the future, training for the CYBATHLON competition should also include stress management and BCI training in noisy and stressful conditions.

All the points above certainly contributed to our results at the actual CYBATHLON competition, where we ended up 5th out of 6. Interestingly enough, except team NITRO 2 (our team was team NITRO 1), which ranked 6th/6, all the other teams already had at least one previous experience in this competition (for the CYBATHLON 2016 and/or the CYBATHLON BCI series 2015) with their pilot, who thus trained to control a BCI for at least 1 year (our pilot trained for 3 months), sometimes much longer. This seems to also suggest that to achieve good BCI design and training control, both BCI scientists teams and BCI CYBATHLON pilots need substantial training and experience, which we lacked for our first CYBATHLON attempt.

## 6. Conclusion

Our results showed a learning at all levels (i.e., user, machine, and experimenters). Indeed, during the few months of training we were able to observe a user learning. Indeed, the classification accuracy significantly increased during training, while our proposed metric significantly TA decreased, both reflecting user learning. In addition, we were able to propose a new Riemannian adaptive approach to reduce EEG distribution shifts within and between sessions. Such an approach could, in the future, be used to improve the user learning by stabilizing the BCI feedback. Controlling a BCI can be long and difficult as generating distinct brain signal that the BCI can recognize is a skill to be learned and we believe that improving the technology to help the user achieve that goal is one of the research area we need to focus on. In addition to the improvement of the technology, our study showed that focus on user training and user experience is also essential. Finally, we also learned a lot by doing mistakes during the training. This enabled us to identify several interesting research directions for the future.

## Data Availability Statement

The raw data supporting the conclusions of this article will be made available by the authors, without undue reservation.

## Ethics Statement

The studies involving human participants were reviewed and approved by Inria COERLE. The patients/participants provided their written informed consent to participate in this study. Written informed consent was obtained from the individual(s) for the publication of any potentially identifiable images or data included in this article.

## Author Contributions

CB, KS, AR, CJ, and FL performed offline data analysis and drafted an initial version of the manuscript. CB, KS, AR, AA, TM, SP, JM, LP, and FL participated in the data acquisition and user training. CB, AR, AA, TM, LP, CJ, and FL contributed to the actual CYBATHLON competition. CJ conducted and analyzed the interview on user experience and acceptability. TM implemented the proposed BCI setup in OpenViBE. FL conceived and supervised the study, data analysis and writing of the manuscript. All authors participated in the technical meetings to design and adapt the BCI system as well as the training procedure. All authors read, edited, and approved the final manuscript.

## Conflict of Interest

The authors declare that the research was conducted in the absence of any commercial or financial relationships that could be construed as a potential conflict of interest.

## Appendix A

**Algorithm 1:** TrainFgMDM

**Input:** *C*_*i* = 1:_*N*__*train*__ : Train SCMs
**Output:**$\tilde{W_{k}}$: Fisher Filters, ${\bar{C}}^{\left(c\right)}$:Means of different classes
1: $\bar{C}=argmin_{C}\overset{i=N_{train}}{\underset{i=1}{\sum }}d_{R}^{2}\left(C,C_{i}\right)$ // (Compute the mean Covariance matrix)
2: $\forall i=1:N_{train},S_{i}=Log_{\bar{C}}\left(C_{i}\right)$ // (Map SCMs to the tangent space, $T_{\bar{C}}$ )
3: $\tilde{W_{K}}=LDA{\left(\left\{vec\left(S_{i}\right)\right\}\right)}_{\left(i=1:N_{train}\right)}$ //vec : vectorize the matrix by stacking the columns of matrix on top of one another // (Compute Fisher Discriminant Filters at $T_{\bar{C}}$, select *K* first filter)
4: $\forall i=1:N_{train},\tilde{S_{i}}=\tilde{W_{K}}{\left({\tilde{W_{K}}}^{T}\tilde{W_{K}}\right)}^{-1}{\tilde{W_{K}}}^{T}vec\left(S_{i}\right)$ // (Filter all training samples at tangent space, $\bar{T_{C}})$
5:$\forall i=1:N_{train},\tilde{C_{i}}=Exp_{\bar{C}}\left(unvec\left(\tilde{S_{i}}\right)\right)$ // (Transfer filtered samples on to the manifold)
6: $\bar{C}=argmin_{C}\overset{i=N_{train}}{\underset{i=1}{\sum }}d_{R}^{2}\left(C,\tilde{C_{i}}\right)$ //(Compute the mean of all filtered train samples)
7: ${\bar{C}}^{\left(c\right)}$ = Train MDM classifier(${\tilde{C}}_{i=1:N_{train}}$) //(Train MDM)

**Algorithm 2:** Test FgMDM

**Algorithm 2:**
**Input:** $C_{i=1:N_{test}}^{{}^{\prime }}$: Test SCMs, ${\bar{C}}^{\left(c\right)}$:Means of different classes, $\tilde{W_{k}}$: Fisher Filters, $\bar{C}$: Mean of train SCMs
**Output:** $y_{i}^{{}^{\prime }}$:Test label
1: $\forall i=1:N_{test},S_{i}=Log_{\bar{C}}\left(C_{i}\right)$ // (Map test SCMs to the tangent space, $T_{\bar{C}}$ )
2: $\forall i=1:N_{test},S_{i}^{{}^{\prime }}=\tilde{W_{K}}{\left({\tilde{W_{K}}}^{T}\tilde{W_{K}}\right)}^{-1}{\tilde{W_{K}}}^{T}vec(S_{i}^{\prime }$) // (Filter all test samples at tangent space, $\bar{T_{C}})$
3: $\forall i=1:N_{test},C_{i}^{{}^{\prime }}=Exp_{\bar{C}}\left(unvec\left({\tilde{S_{i}}}^{{}^{\prime }}\right)\right)$ // (Transfer filtered test samples on to the manifold)
4: $\forall i=1:N_{test},y_{i}^{{}^{\prime }}=argmin_{{\bar{C}}^{\left(c\right)}}d_{R}\left(C_{i}^{{}^{\prime }},{\bar{C}}^{\left(c\right)}\right)$ //(Evaluate test samples by MDM classifier)
